# Evaluating the effect of amoxicillin treatment on the microbiome of *Orbicella faveolata* with Caribbean yellow band disease

**DOI:** 10.1128/aem.02407-24

**Published:** 2025-06-12

**Authors:** Alexi S. Pearson-Lund, Sara D. Williams, Katherine R. Eaton, Abigail S. Clark, Nathaniel Hanna Holloway, Kristen A. Ewen, Erinn M. Muller

**Affiliations:** 1 GEOMAR Helmholtz Centre for Ocean Research 28402, Kiel, Germany; 2 Mote Marine Laboratory, Sarasota, Florida, USA; 3 Cooperative Institute for Marine and Atmospheric Studies, University of Miami 602147 , Miami, Florida, USA; 4 Scouting America, Sea Base, Brinton Environmental Center, Summerland Key, Florida, USA; 5 National Park Service, St. Croix, US Virgin Islands; 6 Scripps Institution of Oceanography, La Jolla, California, USA; Indiana University Bloomington, Bloomington, Indiana, USA

**Keywords:** coral disease, CYBD, antibiotic, amoxicillin, treatment, Base2B, microbiome, *Orbicella faveolata*

## Abstract

Host microbiomes play a key role in coral disease dynamics; thus, it is essential to characterize microbial communities of diseased tissues and identify how they are altered by potential treatments, especially as coral reef populations continue to decline globally. The abundance of *Orbicella faveolata,* a major reef-building Caribbean coral species, has significantly declined due to several stressors, including infectious disease. Caribbean yellow band disease (CYBD) often results in complete colony mortality. We applied an amoxicillin-laced Base2B ointment to CYBD lesion areas on *O. faveolata* within Buck Island Reef National Monument, St. Croix, USVI, trying to halt disease progression. Another CYBD lesion area on the same colony served as a paired untreated control. Microbiomes of the apparently healthy tissue adjacent to the treatment were characterized pretreatment and 2 days post-treatment, along with the paired untreated CYBD controls and nearby healthy colonies. Both microbiomes of untreated CYBD lesions and apparently healthy tissue on CYBD colonies had a significantly higher alpha diversity and significantly differed from those of nearby healthy colonies, suggesting potential systemic effects of CYBD. Amoxicillin treatment significantly changed the microbial community composition of tissues adjacent to the treatment site. The relative abundance of the bacterial family *Vibrionaceae*, a putative pathogen for CYBD and often associated with other coral diseases, was enriched post-treatment. However, the lesion progression rates of treated and untreated lesion areas were similar. Our results suggest that amoxicillin may disrupt the microbiome of adjacent tissue on *O. faveolata*, allowing for opportunistic *Vibrio* sp. bacteria to colonize, and may not be an effective treatment for CYBD.

**IMPORTANCE:**

*Orbicella faveolata*, a primary reef-building coral species in the Caribbean, has been severely impacted by Caribbean yellow band disease. This disease causes tissue loss, which often culminates in the complete loss of the colony since recovery is rarely observed. The present study is significant because the development of an effective long-term treatment for Caribbean yellow band disease and understanding how the microbial partners contribute to pathogenesis are essential for conserving Caribbean coral reefs. While treatment with amoxicillin was not effective, our study uncovered valuable insights into the microbial composition of Caribbean yellow band disease in *O. faveolata*. In addition, this study highlights the possible unintended negative effects of treatment with amoxicillin and casts doubt on *Vibrionaceae* as the culprit of this disease.

## INTRODUCTION

Coral reefs are some of the most diverse and valuable ecosystems on Earth, hosting approximately 25% of known marine species and providing resources for almost a billion people within 100 km of these ecosystems who rely on them for food, livelihoods, and coastal protection (1, 2). The global coverage of living coral has declined by half in the last seven decades due to a variety of factors, including habitat destruction, climate change, overfishing, pollution, and disease (3). Under the current trajectory, 76.8% of corals are predicted to be affected by disease by 2100 (4). Therefore, it is critical to further ascertain the nature of coral disease pathogenesis and develop treatments to curb transmission. The bacterial microbiome is a key piece in the coral disease puzzle as it affects coral health and promotes stress resilience (5).

Coral disease has been a major driver of worldwide coral decline and is thought to be exacerbated by anthropogenic effects, especially climate change (6–8). The coral holobiont is composed of the coral animal, its endosymbiotic zooxanthellae, and its associated microbiome including bacteria, which all interact in complex ways to support holobiont health and function. In order to understand coral disease, it is important to investigate the microbial community composition and to determine how shifts can lead to disease manifestations (9). Coral disease dynamics are complex, and despite there being over 40 described diseases, only six have a known bacterial pathogen that consistently reproduces a disease phenotype (10, 11). The traditional one-pathogen-one disease model has been ineffective in describing coral reef disease dynamics (11), and some evidence suggests that coral disease is associated with a disruption resulting in dysbiosis, which can lead to colonization by opportunistic species, and that this dysbiosis is a hallmark of coral disease (11).

The Caribbean, in particular, is considered a “hotspot” for coral disease activity due to the high prevalence and virulence of several diseases in the region. In fact, this region comprises only 8% of the coral reef area worldwide, but 66% of all diseases and syndromes until 2000 have occurred within Caribbean nations (12–14). A disease of particular concern is Caribbean yellow band disease (CYBD) because it is common and has been a major contributor to the collapse of reef habitats in some areas (15, 16). CYBD was first identified in Florida and has been recorded worldwide, with its classification assigned according to the location where it occurs (15, 17). It was first observed on *Orbicella faveolata,* one of the most important, slow-growing reef-building corals in the Caribbean region (14, 17–19). It causes tissue loss, can impact reproduction, and often culminates in the complete loss of the diseased colony (14, 18, 20). In some reefs, the prevalence of CYBD has been as high as 88% (21). CYBD lesions appear as a translucent yellow band, usually with an area of dead skeleton on one side and apparently healthy tissue on the other, as the lesion expands (22). The rate of tissue loss is about 0.6 cm a month and may take years to fully progress; however, recovery is rarely observed (18).

While much is still unknown about the nature of CYBD, four species of *Vibrio* have been implicated in infection (23). It is suggested that coral-zooxanthellae symbiosis is disrupted first by impairment of the mitotic cell division of zooxanthellae and then by lysis of the symbiont (15). While there has been success in initiating CYBD-like symptoms in corals *in vitro* after exposure to *Vibrio* isolated from CYBD-infected *O. faveolata* in Puerto Rico (24), mechanical transmission was not possible (25), and the spatial pattern of CYBD does not follow a contagious disease model *in situ* (26). Additionally, it has been difficult to indicate the exact cause of disease signs as microbiome studies on CYBD *in situ* using next-generation sequencing techniques have found that distinct bacteria are not consistently present in diseased tissue, and *Vibrio* has been identified often in healthy microbiome samples (27, 28). In Puerto Morelos, Mexico, *O. faveolata* skeletal cores from apparently healthy colonies, apparently healthy tissue from CYBD colonies, and lesion tissue were examined, and *Vibrio* was 2–10 times more abundant in samples from apparently healthy colonies (28). Closek et al. (28) suggest that while some species of *Vibrio* are commensal, it is possible that *Vibrio* has the potential to become pathogenic upon disturbance. The role of *Vibrio* and other bacterial constituents in CYBD is still an active area of inquiry.

In response to increasing coral disease prevalence, there has been growing interest in developing potential treatments. Shading, aspirating, and chiseling a “firebreak” have been tested as possible treatments for *O. favelota* affected by CYBD (29). The firebreak showed initial promise but has not been recommended as a singular solution due to a lack of long-term success (29). Since there is some evidence that pathogenic bacteria are the mechanistic cause of CYBD, treatment with antibiotics may prove effective (23). Antibiotic treatments have been utilized to treat other coral diseases with varying success (30–35). Administration of amoxicillin mixed into a topical ointment termed Base2B (Ocean Alchemists LLC) has effectively halted the progression of stony coral tissue loss disease (SCTLD; 32, 34, 35), and administration of ampicillin and paromomycin arrested white band disease in laboratory trials (30, 31). Additionally, application of ointment containing naturally produced antibacterial and antiviral active ingredients was effective in treating another lethal coral disease, black band disease (36). Thus, ointments with antibiotics or antibacterial ingredients may be effective at treating other coral diseases.

The role that the bacterial partners in the coral holobiont play in coral disease transmission and susceptibility requires further research in order to develop and test mitigation techniques. Although antibiotic treatments for several coral diseases have been tested (32) and successfully integrated into management practices in the case of SCTLD (37), little is known about the effect that these applications may have on the coral microbiome. Additionally, antibiotic treatments for CYBD, a major contributor to Caribbean reef decline, have not been tested previously. Thus, we investigated three objectives: i) evaluate potential differences in microbial communities among tissue from healthy control colonies and both lesion and apparently healthy tissues from CYBD-affected colonies; ii) determine if amoxicillin treatment halts CYBD progression; and iii) decipher how amoxicillin treatment alters the microbiome of *O. faveolata* colonies with CYBD.

## MATERIALS AND METHODS

### Study location, antibiotic treatment application, and monitoring

All fieldwork for this study was carried out under permits VICR-2021-SCI-0003 and VIIS-2021-SCI-0015 at Buck Island Reef National Monument, St. Croix, USVI (17.788N, 64.598 W), from June 2020 to January 2021. Healthy *Orbicella faveolata* colonies (*n* = 5) and those affected with CYBD (*n* = 8) were identified and tagged with a unique colony number. Diseased colonies had at least two CYBD lesions present. On each diseased colony, one lesion area was designated for treatment with amoxicillin (i.e., treated lesion) and another was designated as the paired untreated control (i.e., untreated lesion). Due to the constraints of the study design requiring two lesions on each colony and the endangered status of the corals, the sample size was limited. However, the number of colonies sampled was similar to that observed in previous *in situ* studies of coral disease microbiomes (e.g., 28).

**Fig 1:**
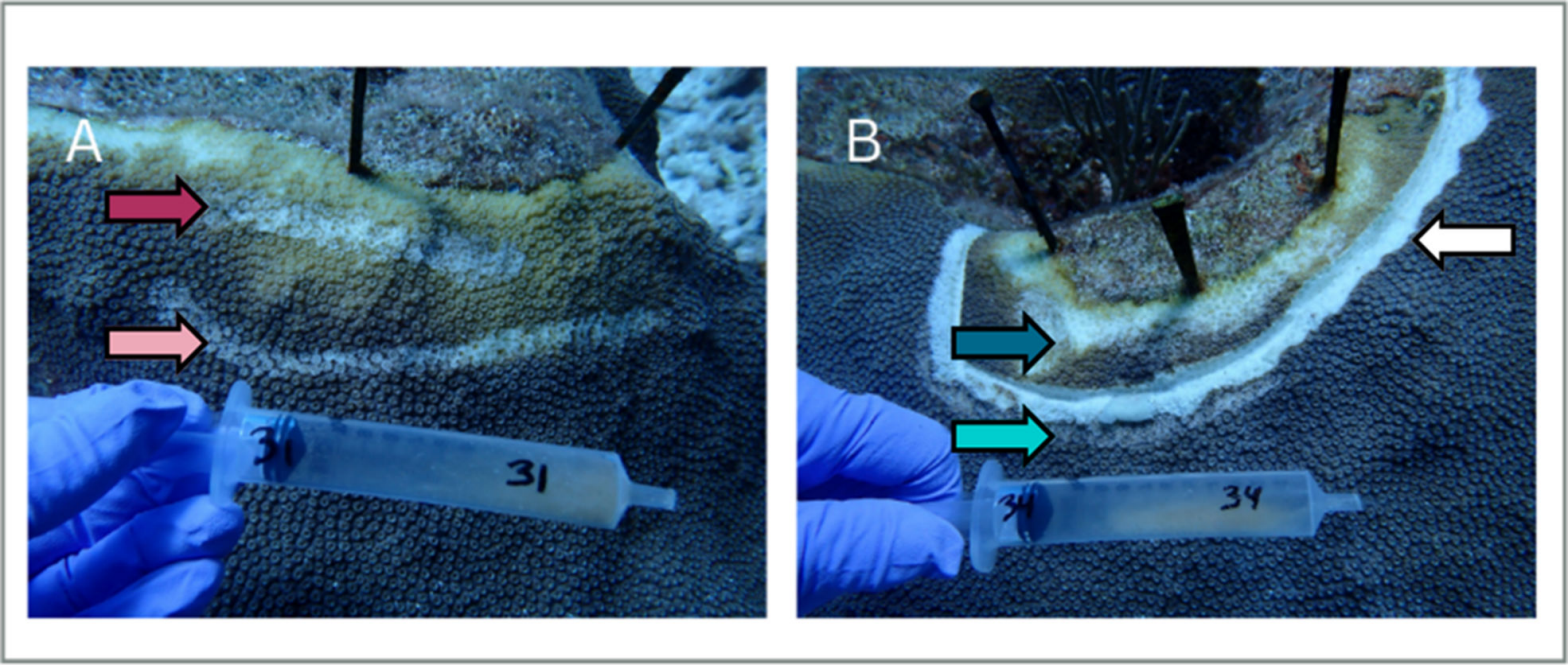
Photo of the sampling design of (A) an untreated lesion area or (B) an amoxicillin-treated lesion area. A syringe was used to collect a slurry of mucus and tissue. Caribbean yellow band disease lesions are yellow, and the sampling location was visualized by an arrow color-coded to denote treatment and tissue type. (**A**) Sampling of the lesion tissue (maroon arrow) and apparently healthy tissue (pink arrow) from an untreated lesion area. (**B**) “Firebreak” 1 cm x 1 cm with amoxicillin-laced ointment in the groove (white arrow) between the lesion tissue (dark blue arrow) and the apparently healthy tissue (light blue arrow). All apparently healthy tissue was sampled approximately 10 cm away from the lesion. Note the three nails positioned above the lesion to monitor lesion progression.

Prior to treatment with amoxicillin, a firebreak was created around the treated lesion area using an underwater angle grinder (Nemo Power Tools, Las Vegas, NV, USA). The firebreak was approximately 1 cm wide and 1 cm deep with approximately 1 cm of apparently healthy tissue between the lesion edge and the firebreak to serve as a buffer, following the method described in (29). Additionally, Randall et al. (29) showed a relatively rapid mortality of the diseased tissue (i.e., lesion area) once isolated by trenching. Therefore, the concept applied to promote treatment efficacy was to isolate the diseased area, cause rapid mortality of the diseased tissue, and treat ahead of the disease margin into the apparently healthy tissue with amoxicillin to potentially remove pathogenic agents. The antibiotic amoxicillin (PhytoTech Labs, Lenexa, KS, USA) was mixed with Base2B (Ocean Alchemists LLC), in an 8:1 Base2B-to-amoxicillin ratio by weight, less than 6 hours before application to create an ointment following the methodology outlined in (32). The ointment allowed for a regulated release of amoxicillin over 3 days. Using a syringe, the treatment was applied to the diseased coral along the groove of the firebreak and over the lip into the apparently healthy tissue (Fig. 1).

Disease progression was monitored at 1 (July 2020), 3 (October 2020), and 6 (January 2021) months post-treatment. In order to measure the progression of the disease after treatment, three nails were placed equidistant from one another at the top of each lesion. Disease progression at each time point was assessed by measuring the distance from each nail to the apparently healthy tissue beyond the firebreak *in situ*. Each lesion was measured in triplicate at each time point using three nails as markers. However, two lesions near the “untreated lesion” areas had nails that became dislodged. These and their corresponding replicates for the “treated lesion” areas were therefore removed from the analysis. Photos were taken both pre- and post-treatment and at each lesion progression time point as documentation (Olympus Tough TG-6, Bethlehem, PA, USA).

### Sample collection

Samples for microbial analysis were collected from the five healthy *O. faveolata* colonies and from the paired treated and untreated lesion areas of the five colonies with CYBD at two time points: pretreatment on 22 June 2020, and 2 days post-treatment on 24 June 2020. A sterile 10 mL syringe was used to collect each sample by scraping over several polyps while pulling the plunger to collect a slurry of coral mucus and tissue. Initial samples were taken approximately 10 cm from the base of each monitored lesion into the apparently healthy tissue for both untreated (Fig. 1A, pink arrow) and treated lesion areas before applying the treatment (Fig. 1B, light blue arrow). At the post-treatment time point, samples were collected again from a similarly adjacent area for the untreated and treated CYBD lesion areas (Fig. 1B, pink and light blue arrow respectively). Additionally, samples were collected from directly within the lesion tissue next to the untreated (Fig. 1A, maroon arrow) and treated lesion areas (Fig. 1B, dark blue arrow). Coral colonies (*n* = 5) with no signs of disease were sampled at both time points to serve as healthy controls. Thus, this sampling scheme resulted in three types of coral tissue and mucus slurry samples: healthy control, apparently healthy tissue from diseased colonies, and lesions. All sample types, except for lesions, were collected at both the pre-and post-treatment time points. Samples were flash-frozen in liquid nitrogen in the field and then stored at −80°C until the additional processing steps described below.

### Sample processing and sequence analysis

DNA was isolated from the coral mucus and tissue slurry samples using the DNeasy PowerSoil Kit (QIAGEN, Germantown, MD, USA) and the manufacturer’s protocol with modifications (following reference 38). DNA quality and concentrations were measured with a NanoDrop One Microvolume UV-Vis Spectrophotometer (Thermo Fisher Scientific, Waltham, MA, USA). DNA isolates were barcoded, amplified, and sequenced by MR DNA (Shallowater, TX, USA). Amplification of the 16S rRNA gene variable region (V4) was performed utilizing primers 515F (GTGYCAGCMGCCGCGGTAA [39]) and 806R (GGACTACNVGGGTWTCTAAT; Archaea 806R [40]). A 30-cycle polymerase chain reaction (PCR) was performed using the HotStarTaq Plus Master Mix Kit (QIAGEN, Germantown, MD, USA) under the following conditions: 95°C for 5 min, followed by 30 cycles of 95°C for 30 s, 53°C for 40 s, and 72°C for 1 min, and by a final elongation step at 72°C for 10 minutes. Amplification success was determined by running PCR products on a 2% agarose gel. Samples were pooled together in equal proportions based on their molecular weight and DNA concentrations. Pooled samples were purified using calibrated Agencourt Ampure XP beads (Beckman Coulter, Inc., Brea, CA, USA). Sequencing was performed on an Illumina MiSeq following the guidelines of the manufacturer.

Sequence data processing and analyses were performed in the program R (4.3.2 [41]). A total of 38,659,556 reads across 58 samples were processed using the DADA2 pipeline (v1.16 [42]) in R (see Table S1 for the number of reads retained at each step). Upon inspection of the quality plot, forward and reverse reads were truncated to 190 base pairs at the 3′ at the first position where the quality read score was ≤2. Reads with the presence of N or a total expected error of >2 were discarded. This resulted in a total of 23,587,579 reads. An initial total of 39,975 amplicon sequence variants (ASVs) were determined from unique reads, and paired-end reads were merged. ASVs that did not match a target length of 250–255 (13,873 ASVs) were discarded. Two-parent chimeras were removed (16,308 ASVs), and taxonomy was assigned to 100% sequence identity using the Silva reference database (v138.1) to preserve the high resolution of ASV data (43). An average of 92.46% of initial reads, corresponding to 9,794 ASVs, were retained through the quality filtering pipeline. The ASV table resulting from DADA2 processing was imported into phyloseq (v1.44.0) (44). All zero taxa and taxa in less than four samples were removed, and 9,478 ASVs (21,032,447 reads) were left remaining. A total of 659 ASVs taxonomically identified as chloroplasts or mitochondria sequences were removed (corresponding to 2,958,698 and 327,050 reads, respectively), which resulted in 8,819 ASVs (17,746,699 reads). The phyloseq object was then further subsetted to exclude six samples (three lesions and three apparently healthy tissue samples) that were treated with the antibiotic gentamicin. These samples were collected alongside the amoxicillin-treated data set and therefore needed to be included in the bioinformatics processing, but were omitted from this study due to low sample size, which resulted in a lack of statistical power. The above processing steps resulted in a final number of 8,819 ASVs (13,207,743 reads).

### Statistical analysis

Disease progression rates between treatments and their respective controls were first checked for normality using a Shapiro-Wilks test. Then a two-tailed paired *t*-test was performed to determine if the disease progressed more so when treated or when left untreated. A paired *t*-test was chosen as amoxicillin-treated and untreated lesion areas were on the same colonies.

To evaluate differences in the microbial communities among the different tissue types and when treated with amoxicillin, the beta diversity was calculated using the vegan package (version 2.6–4 [45]). First, the entire data set was compared, and then appropriate subsets of the samples were analyzed further in relation to our main objectives: (i) untreated tissue types (healthy control, apparently healthy, and lesion) at the “post-treatment” time point in order to identify baseline differences among the microbial communities of tissue from healthy and diseased colonies; (ii) only apparently healthy tissue (i.e., adjacent to the antibiotic treatment) from diseased colonies and healthy controls to assess changes pre- and post-treatment, as well as between treated and untreated coral tissue; and (iii) lesion tissue to decipher if there were differences between microbial communities of treated and untreated corals with CYBD. Bray-Curtis dissimilarity distances (vegan package [45]) were used to quantify differences between samples in the beta-diversity statistical tests. Permutational multivariate analysis of variance (PERMANOVA) was used to identify significant differences between the three untreated tissue types (healthy control, apparently healthy, and lesion) or treatments (untreated, amoxicillin, and healthy control) at the post-treatment time point. In addition, PERMANOVAs were used to assess significant differences between pre- and post-treatment time points. All PERMANOVAs were performed using the adonis2 function in the R package vegan (45). Homogeneity of group dispersion using the betadisper function was assessed, and a *post-hoc* Tukey Honestly Significant Difference (HSD) test was used to determine significant differences in the multiple comparisons. Non-metric multidimensional scaling (NMDS) analyses were utilized to assess similarities in bacterial communities.

Alpha diversity as a function of tissue type, treatment, and time point was assessed using two different metrics: species richness and Shannon diversity. All alpha diversity metrics were calculated using the vegan package (45). The Shapiro-Wilks normality test was then used to assess if the normality assumption of an analysis of variance test (ANOVA) was met. Upon meeting assumptions, ANOVAs and *post-hoc* Tukey HSD tests were used to determine significance.

Differentially abundant microbial taxa as a function of tissue type and time point were identified using the R package corncob, which uses a beta-binomial model that allows for overdispersion in the taxon’s counts to be associated with covariates of interest (46). A limitation of corncob is its inability to detect perfectly separating taxa found only in one group (47). To account for this, the bottom quartile of the reads was calculated on the entire data set to determine an appropriate pseudo count to add to all samples. The data were subsetted to either include the untreated tissue types at the post-treatment time point or the apparently healthy tissue pre- and post-treatment with amoxicillin. The relative abundance of ASVs was then calculated, and the top 100 relatively abundant taxa were identified for both data subsets. Corncob was then used to determine which ASVs in the top 100 relatively abundant taxa were differentially abundant. We used bubble plots to display the relative abundance of significant ASVs glommed by family as a function of tissue types and time points. ASVs were glommed due to the low taxonomic resolution of the data set as the vast majority of ASVs could not be identified beyond the family level. Post-filtration, the data set was composed of 8,819 ASVs, with an average read depth of 2,012.

## RESULTS

### Microbial composition differed between healthy controls and apparently healthy tissue on untreated CYBD colonies, but was similar between apparently healthy and lesion tissue on CYBD colonies

First, the entire data set was compared, and bacterial communities were found to be significantly different between healthy control corals, untreated lesion areas, and treated lesion areas (PERMANOVA: df = 2, F = 2.27, *P* = 0.001; Table S2). Samples collected initially and 2 days later also had significantly different bacterial communities (PERMANOVA: df = 1, F = 1.827, *P* = 0.032; Table S2); however, the interaction of treatment and time was not significant (Table S2).

**Fig 2:**
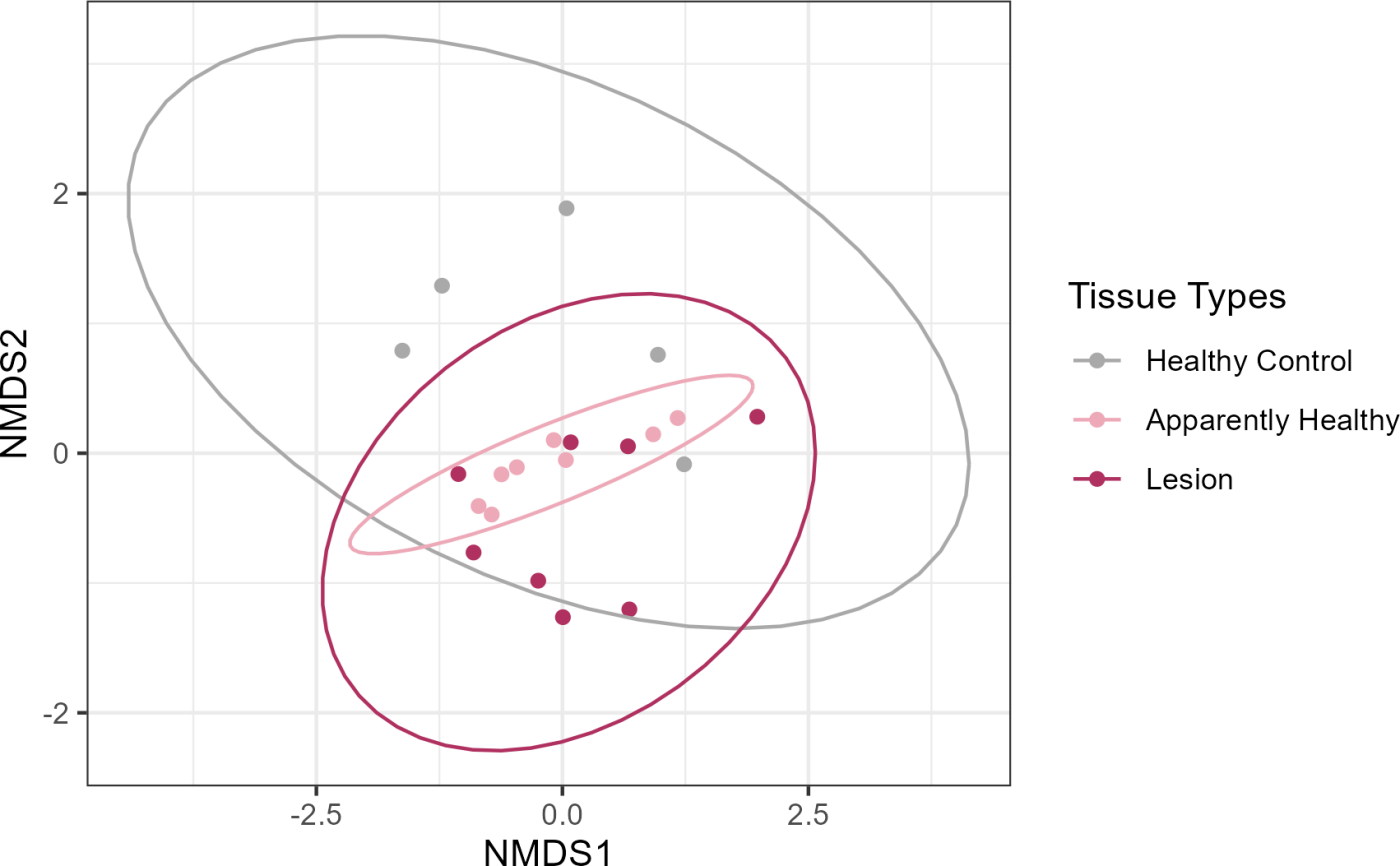
Non-metric multidimensional scaling analysis (NMDS) of the Bray-Curtis distances calculated on the amplicon sequence variants (ASVs) of untreated tissue types at the post-treatment time point. Healthy control (gray) colonies had no signs of Caribbean yellow band disease (CYBD). From CYBD colonies, apparently healthy tissue (pink) was sampled approximately 10 cm beyond a CYBD lesion, while the lesion tissue (maroon) was sampled within the CYBD lesion. Only samples at the post-treatment time point were included in this analysis in order to keep the data set balanced since lesion tissue was only sampled at that time point. None of the included samples were treated with antibiotics. Points represent samples collected from the following tissue types: healthy control (*n* = 5), apparently healthy (*n* = 8), and lesion (*n* = 8). Ellipses represent the 95% confidence interval. Stress was 0.171.

Significant differences in the beta diversity were determined between healthy control colonies and untreated CYBD-affected colonies (PERMANOVA: df = 2, F = 1.548 *P* = 0.031; Table S2). Specifically, bacterial communities of healthy control corals and apparently healthy tissue on CYBD corals were significantly different (Fig. 2; multiple comparison adjusted *P* = 0.045, Table S2). Interestingly, the bacterial communities of the healthy control and CYBD lesion tissue did not significantly differ (multiple comparison adjusted *P* = 0.198, Table S2). However, the NMDS analysis showed these two tissue types largely separated in ordination space (Fig. 2), which suggests trends toward differentiation, but the sample size may have been too low to detect significant differences using PERMANOVA. The bacterial communities of apparently healthy tissue on CYBD corals and CYBD lesion tissue also did not significantly differ (Fig. 2; multiple comparison adjusted *P* = 0.801, Table S2).

### Alpha diversity was significantly reduced, and microbial profiles were distinct in healthy controls compared to apparently healthy tissue on untreated CYBD colonies and lesion tissue

**Fig 3:**
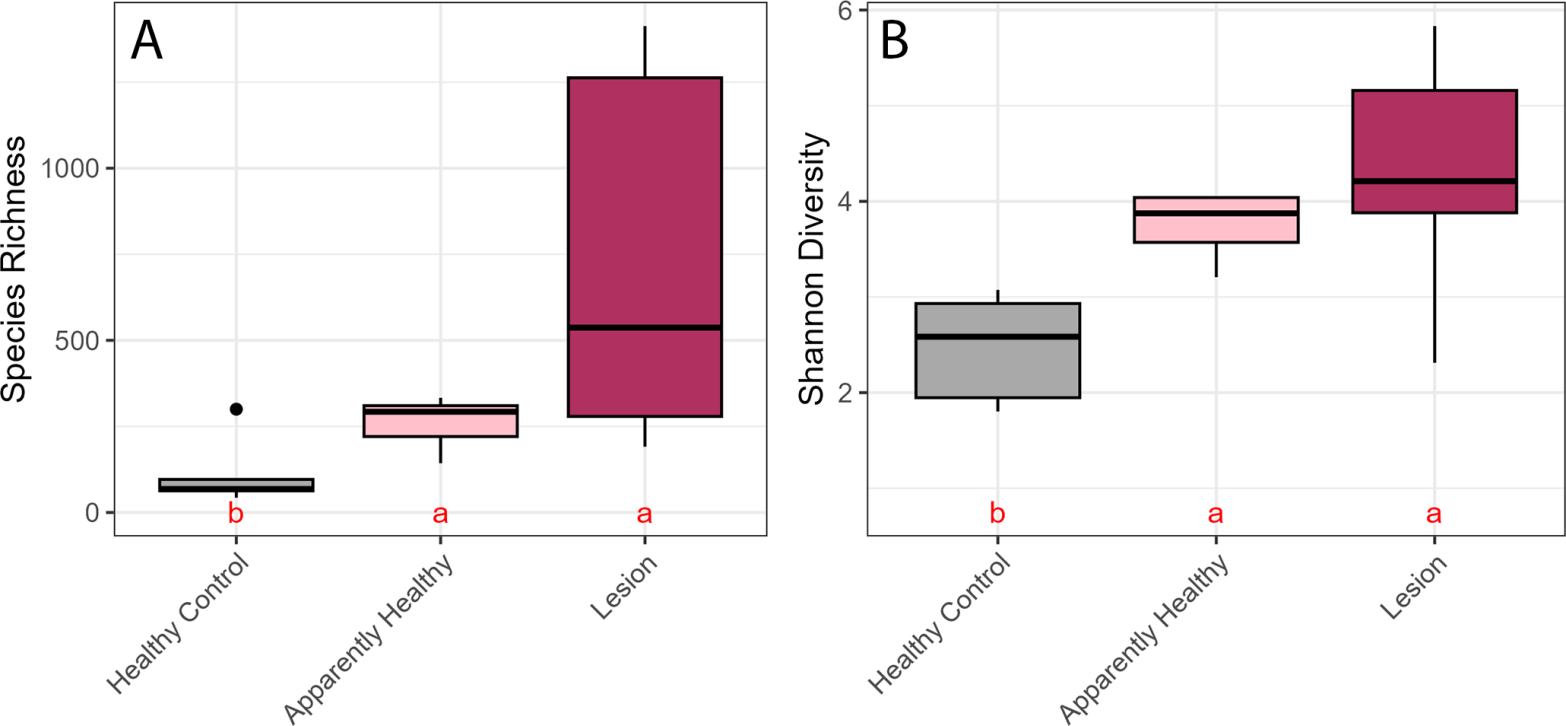
Alpha diversity of the untreated tissue types measured by (A) species richness and (B) Shannon diversity. Letters denote significant differences determined by *post-hoc* Tukey Honestly Significant Difference test. Healthy control (gray) colonies had no signs of Caribbean yellow band disease (CYBD). From CYBD colonies, the apparently healthy tissue (pink) was sampled approximately 10 cm beyond a CYBD lesion, while the lesion tissue (maroon) was sampled within the CYBD lesion.

Significant differences in both species richness (ANOVA: df = 2, F = 11.7, *P* = 0.00056; Table S3) and Shannon diversity (ANOVA: df = 2, F = 8.48, *P* = 0.0025; Table S3) were determined among healthy control, untreated apparently healthy, and untreated lesion tissue samples. Alpha diversity of the bacterial communities was significantly lower within the healthy control tissue compared with the apparently healthy tissue on untreated CYBD-affected colonies (Fig. 3; richness, Tukey *post-hoc* comparison *P* = 0.028; Shannon, Tukey *post-hoc* comparison *P* = 0.029; Table S3) as well as the untreated lesion tissue (Fig. 3; richness, Tukey *post-hoc* comparison *P* = 0.0003; Shannon, Tukey *post-hoc* comparison *P* = 0.002; Table S3). However, the alpha diversity within the apparently healthy tissues and the lesion tissues of untreated CYBD colonies did not significantly differ (Fig. 3; richness, Tukey *post-hoc* comparison *P* = 0.084, Shannon, Tukey *post-hoc* comparison *P* = 0.332; Table S3).

**Fig 4:**
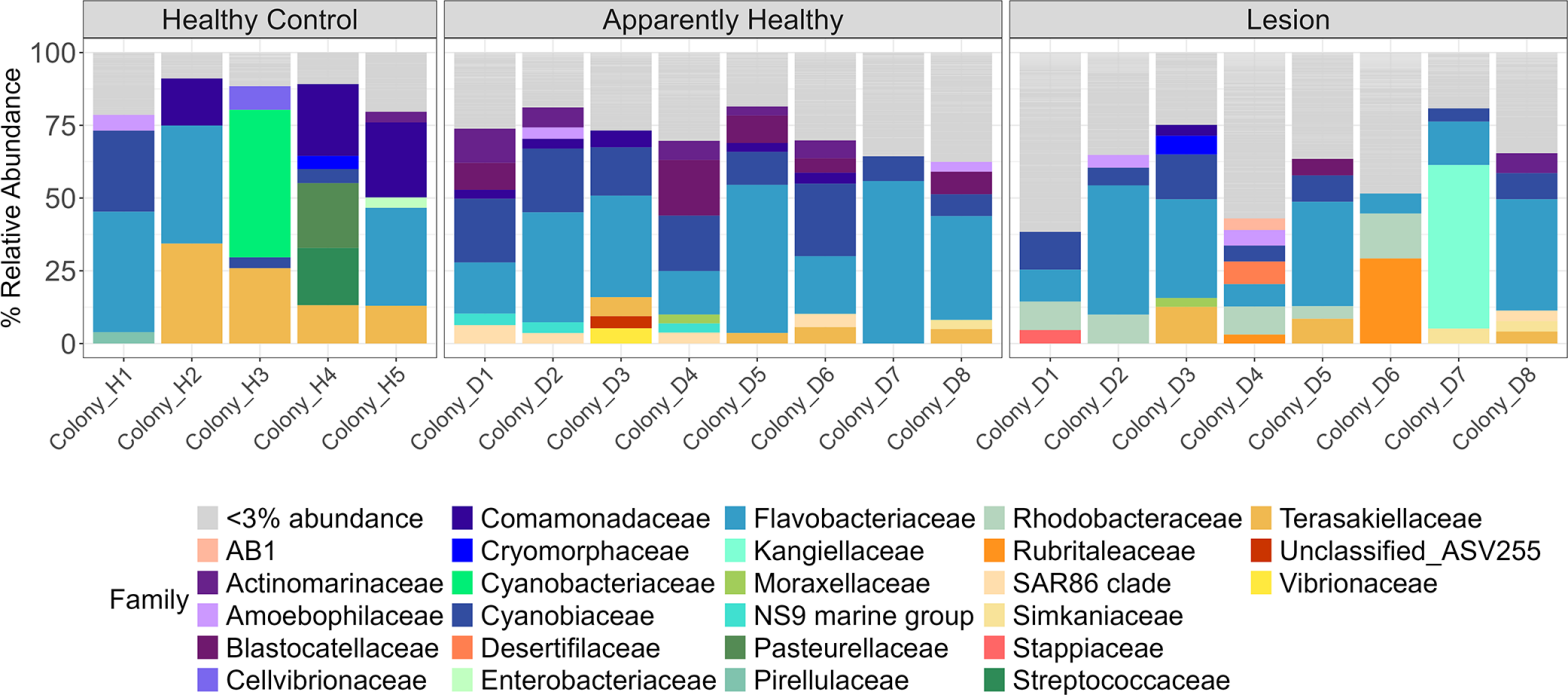
Relative abundance of bacterial families in the untreated tissue type samples. Healthy control colonies had no signs of CYBD. Apparently healthy tissue was collected from CYBD colonies approximately 10 cm beyond a CYBD lesion, while the lesion tissue was sampled within the CYBD lesion. Only samples at the post-treatment time point were included in this analysis since the lesion tissue was only sampled at that time point. None of the included samples were treated with antibiotics. Displayed are families with a relative abundance over 3%. Families with relative abundance under 3% were grouped and represented in gray.

**Fig 5:**
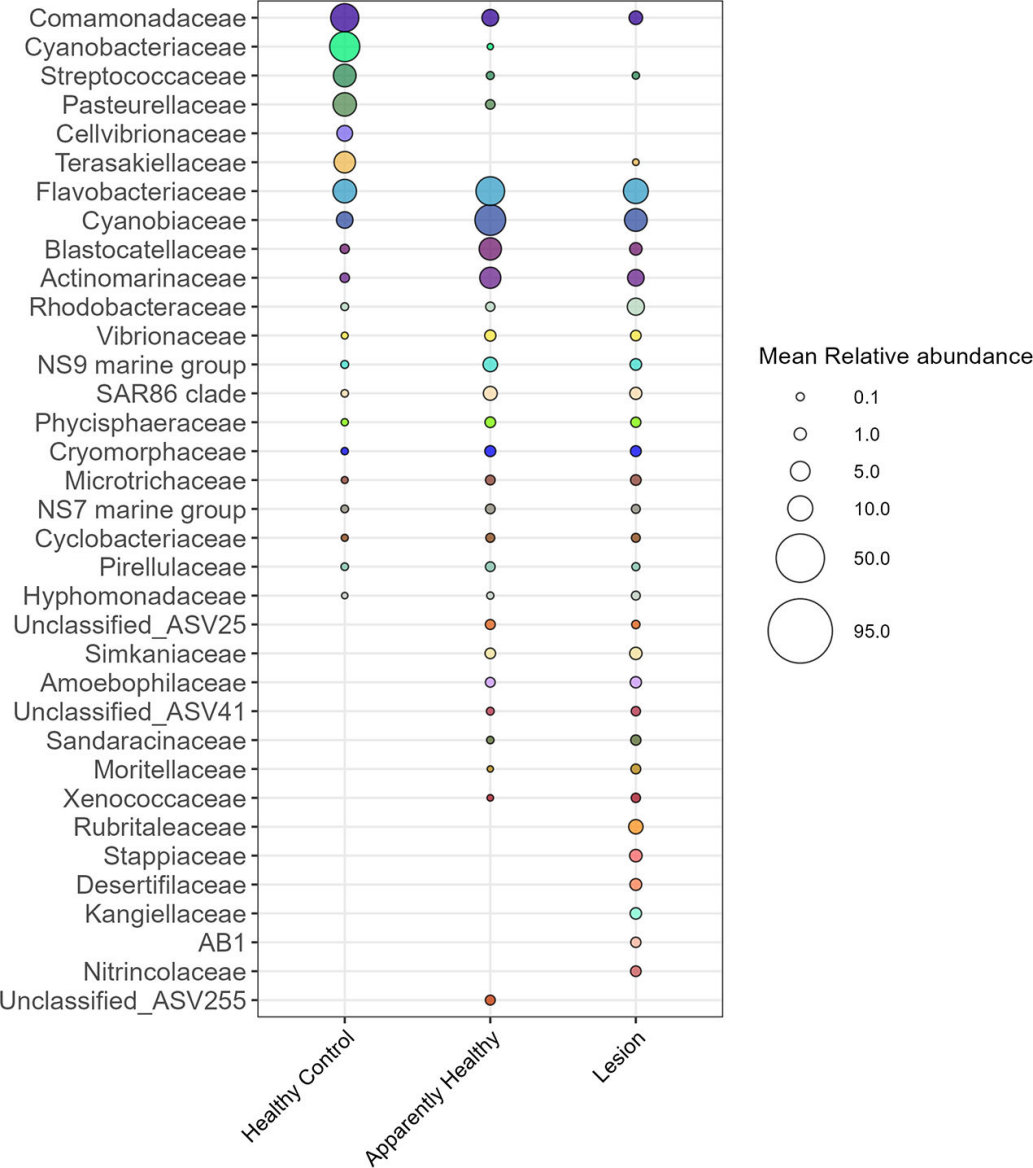
Differentially abundant amplicon sequence variants (ASVs) glommed by family and their relative abundances in the different tissue-type samples**.** Relative abundance was calculated for all of the ASVs in the untreated tissue-type data subset, and the top 100 abundant taxa were identified. Significant ASVs of the top 100 abundant taxa were determined by corncob analysis between each tissue type. Differentially abundant ASVs were glommed by family, due to the low resolution at lower taxonomic ranks. Healthy control colonies had no signs of Caribbean yellow band disease (CYBD). Apparently healthy tissue was collected from CYBD colonies approximately 10 cm beyond a CYBD lesion. The lesion tissue was within the CYBD lesion. Only samples at the post-treatment time point were included in this analysis since the lesion tissue was only sampled at that time point. None of the included samples were treated with antibiotics.

The five most relatively abundant bacterial families in the healthy controls (mean ± standard error) were *Flavobacteriaceae* (23.38% ± 9.39%), *Terasakiellaceae* (17.67% ± 5.63%), *Comamonadaceae* (13.33% ± 5.69%), *Cyanobiaceae* (7.90% ± 5.05%), and *Streptococcaceae* (3.97% ± 3.93%) (Fig. 4). More ASVs were significantly enriched in tissue from CYBD colonies than tissue from healthy controls (Fig. 5). There were 43 significantly different ASVs between healthy controls and apparently healthy tissue on CYBD colonies, with 29 ASVs enriched in the latter and 14 ASVs in the former (Fig. S1). There were 41 significantly different ASVs between healthy controls and lesion tissue, with 28 ASVs in higher abundance in the latter and 13 ASVs in the former (Fig. S2). ASVs that were significantly positively enriched in the healthy controls belonged to just eight families (Fig. 5; Fig. S1 and S2).

The five most relatively abundant bacterial families in the apparently healthy tissue of CYBD colonies were (mean ± standard error) *Flavobacteriaceae* (33.43% ± 5.37%), *Cyanobiaceae* (16.49% ± 2.34%), *Blastocatellaceae* (6.69% ± 2.22%), *Actinomarinaceae* (5.03% ± 1.24%), and *Comamonadaceae* (3.40% ± 0.37%). The bacterial communities of the lesion tissue were dominated by *Flavobacteriaceae* (24.12% ± 5.46%)*, Cyanobiaceae* (8.17% ± 1.53%)*, Rhodobacteraceae* (6.77% ± 1.83%)*, Rubritaleaceae* (4.49% ± 3.56%)*,* and *Terasakiellaceae* (4.24% ± 1.49%) (Fig. 4). There were 29 ASVs that significantly differed between apparently healthy and lesion tissues on CYBD colonies, with 12 ASVs enriched in the apparently healthy tissue and 17 in the lesion tissue (Fig. S3). Fourteen families that were differentially abundant were not present in the healthy control tissue but were in the lesion tissue, with most of these unique families only being present in the lesion tissue. In addition, putative pathogens such as *Rhodobacteraceae* and *Vibrio* were enriched in the lesion tissue compared to healthy controls (Fig. 5).

### Amoxicillin treatment did not effectively halt CYBD lesion progression

**Fig 6:**
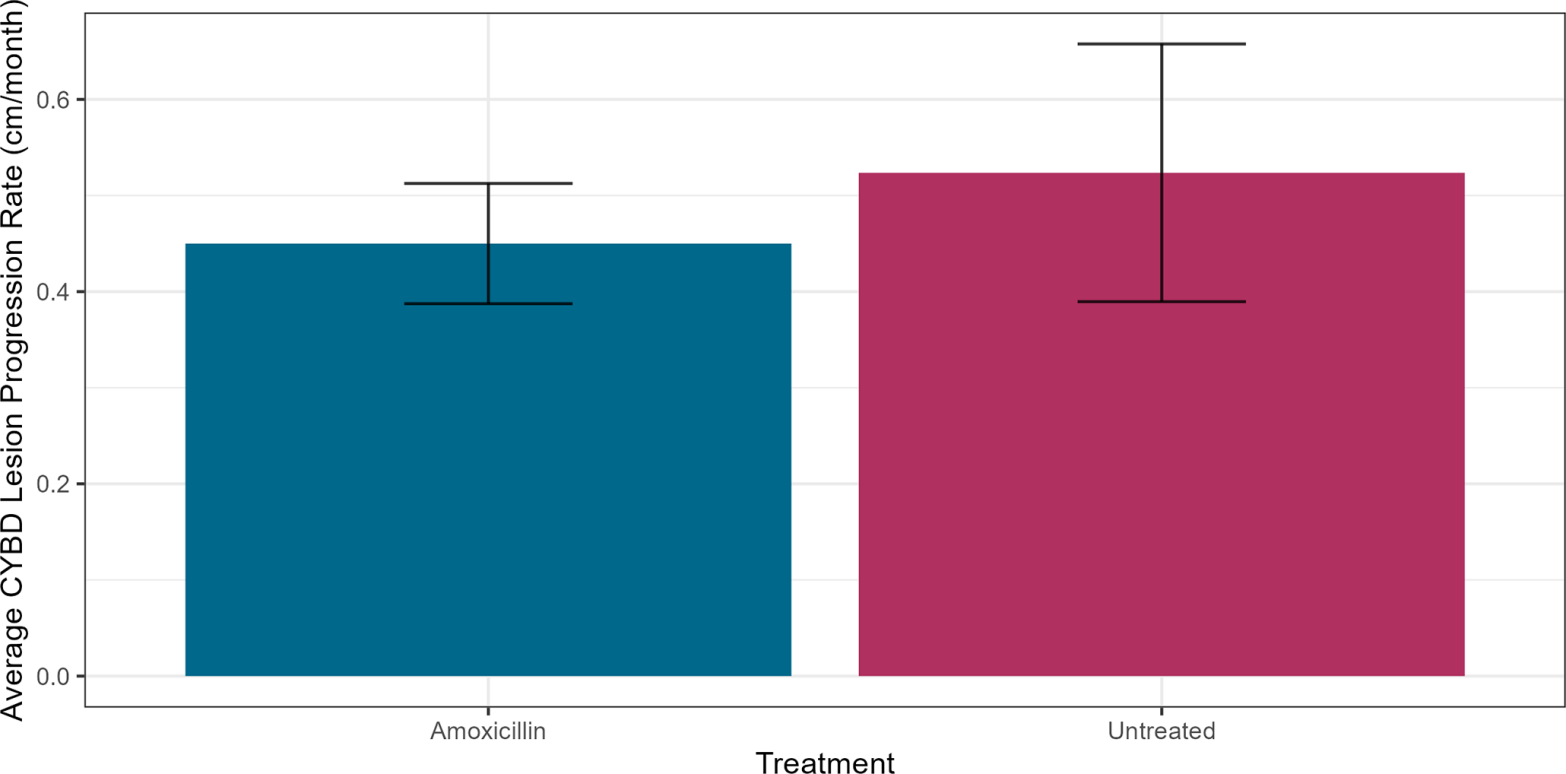
Average disease progression (cm/month) of Caribbean yellow band disease (CYBD) on *Orbicella faveolata* treated with amoxicillin. The average disease progression rate (centimeter per month) was calculated over 6 months by measuring the average distance from three nails, marking the origin of the lesion, to the apparently healthy margin. A paired sample *t*-test was used to determine that there were no significant differences in the average progression rates of lesions near amoxicillin-treated areas (blue) and lesions near untreated areas (maroon) (*P* = 0.519). Error bars represent the standard error of the mean.

A pairwise *t*-test indicated that there was no difference in the average disease progression rates of CYBD lesions from lesion areas that were treated with the antibiotic amoxicillin and Base2B ointment compared to the untreated tissues (t = 0.666, *P* = 0.519, df = 11). The average disease progression for lesions from treated lesion areas was 0.45 ± 0.063 cm/month, while the progression of lesions from untreated areas was 0.52 ± 0.134 cm/month (Fig. 6).

### Treatment with amoxicillin significantly changed the microbial community composition within the apparently healthy tissue but not within the CYBD lesion tissue

**Fig 7:**
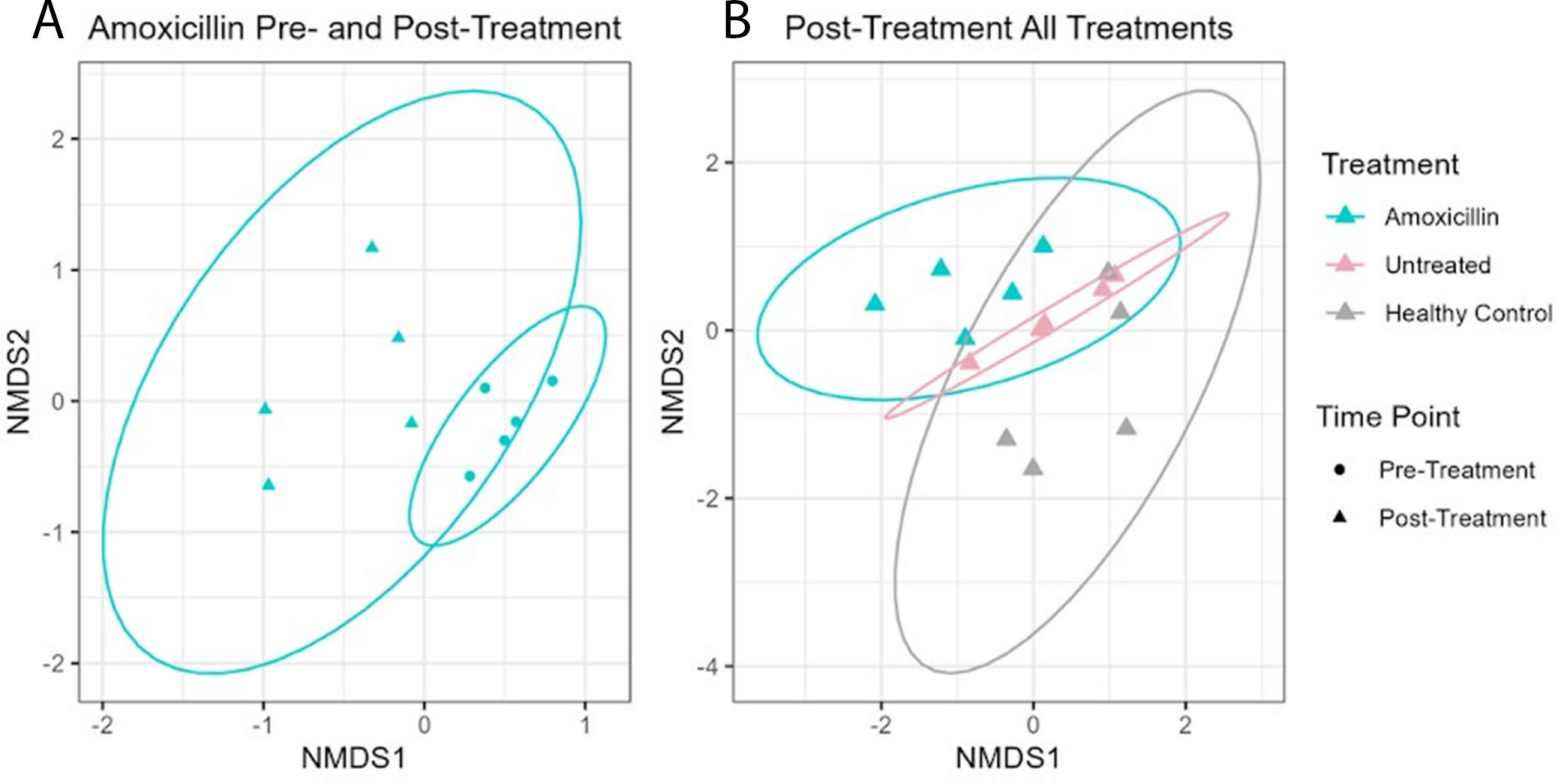
Non-metric multidimensional scaling (NMDS) analysis of the Bray-Curtis distances calculated on the bacterial amplicon sequence variants (ASVs) of either (A) apparently healthy tissue pre- (circles) and post-treatment (triangles) with amoxicillin or (B) apparently healthy tissue post-treatment with amoxicillin (blue), untreated apparently healthy tissue (pink), and healthy controls (gray). Healthy control (gray) colonies had no signs of Caribbean yellow band disease (CYBD). Untreated apparently healthy tissue (pink) was tissue on CYBD colonies that appeared healthy, was 10 cm beyond a CYBD lesion, and was left untreated. Amoxicillin-treated tissue (blue) was tissue on CYBD colonies that appeared healthy, was 10 cm beyond a CYBD lesion, and had been treated with amoxicillin at the post-treatment time point. NMDS of the Bray–Curtis distances for (**A**) pre- and post-treatment with amoxicillin (stress = 0.098). (**B**) Post-treatment with either amoxicillin or untreated and healthy control (stress = 0.151). Points represent samples, and *n* = 5 for all treatments. Ellipses represent the 95% CI.

**Fig 8:**
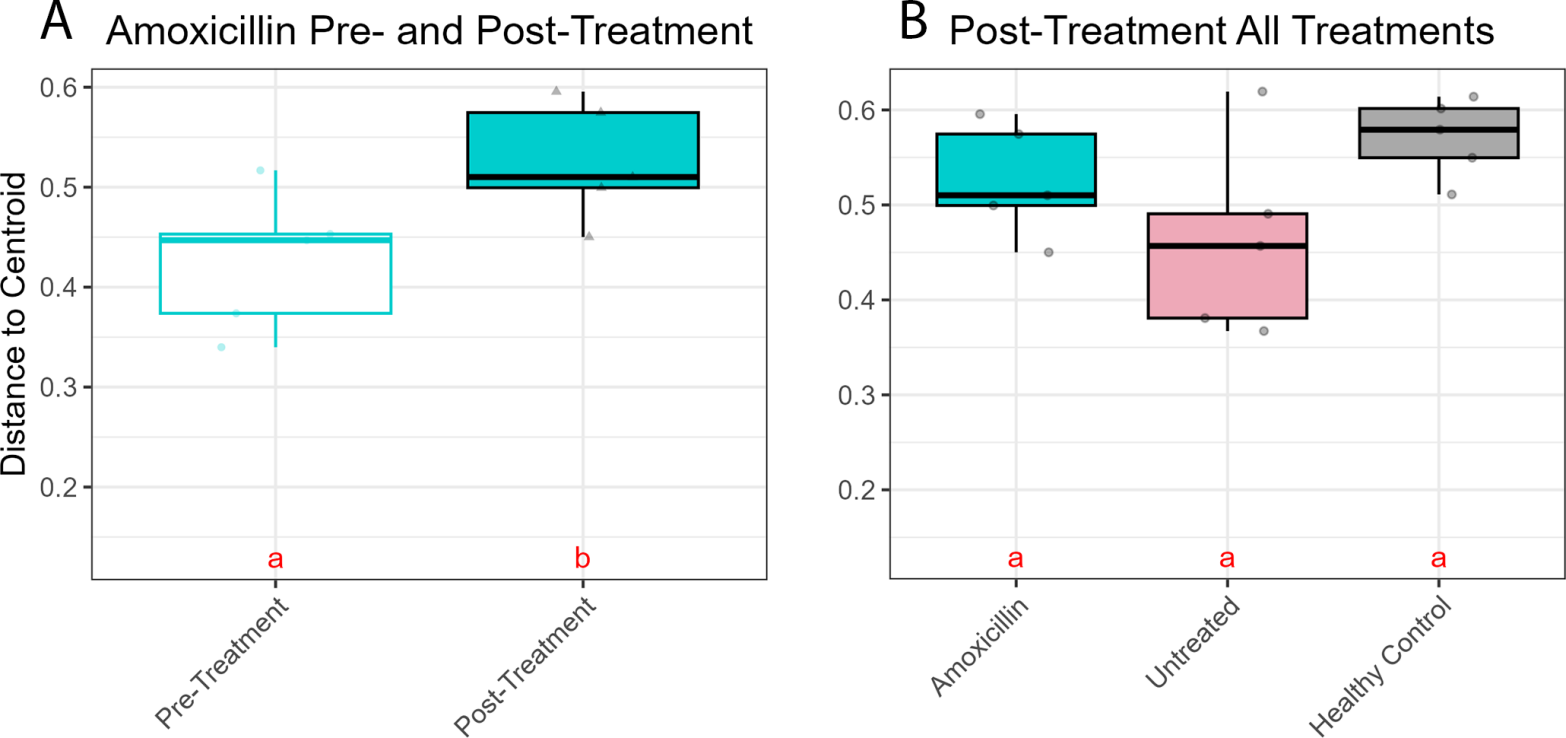
Betadisper of (A) apparently healthy tissue pre- (white) and post-treatment (blue) with amoxicillin and (B) apparently healthy tissue post-treatment with amoxicillin (blue), apparently healthy untreated (pink), or healthy controls (gray). Healthy control (gray) colonies had no signs of Caribbean yellow band disease (CYBD). Untreated apparently healthy tissue (pink) was tissue on CYBD colonies that appeared healthy, was 10 cm beyond a CYBD lesion, and was left untreated. Amoxicillin-treated apparently healthy tissue was tissue on CYBD colonies that appeared healthy, was 10 cm beyond a CYBD lesion, and had been treated with amoxicillin at the post-treatment time point. Letters denote significant differences determined by a *post-hoc* Tukey Honestly Significant Difference pairwise comparison test.

Microbial communities of apparently healthy tissue on CYBD corals significantly differed pre- and post-amoxicillin treatment (Fig. 7A, PERMANOVA: df = 1, F = 2.441, *P* = 0.008; Table S2). The observed difference in beta diversity between pre- and post-treated apparently healthy tissue was further explained by a multivariate homogeneity of group dispersion (variances) analysis, which revealed increased dispersion after treatment (Fig. 8A; *P* = 0.040; Table S4). However, microbial communities of the lesion tissue did not significantly differ between treatments (Fig. S4, PERMANOVA: df = 1, F = 1.143, *P* = 0.235; Table S2). At the post-treatment time point, microbial communities of the non-lesion samples (apparently healthy tissue treated with amoxicillin, apparently healthy tissue untreated, and healthy controls) significantly differed among treatments (Fig. 7B; PERMANOVA: df = 2, F = 1.695, *P* = 0.014, Table S4). Multiple comparison tests determined that the microbial communities of apparently healthy tissue treated with amoxicillin did not significantly differ from those of the untreated apparently healthy tissue (Fig. 7B; *P-*adjust = 0.129; Table S2). However, microbial communities of healthy control and treated apparently healthy tissues were significantly different post-treatment (Fig. 7B; *P-*adjust = 0.009; Table S2). These results were supported by the NMDS analysis (Fig. 7B). Betadisper analysis showed no significant difference in dispersion between healthy controls and apparently healthy tissues post-treatment (Fig. 8B; Table S4). Additionally, there were no notable significant differences in bacterial communities between time points for the untreated apparently healthy tissue (df = 1, F = 0.577, *P* = 0.882; Table S2) or for the healthy controls (df = 1, F = 0.486, *P* = 0.937; Table S2).

### Alpha diversity was similar among all treatments and time points, but Vibrionaceae abundance increased in apparently healthy tissue post-treatment with amoxicillin

**Fig 9:**
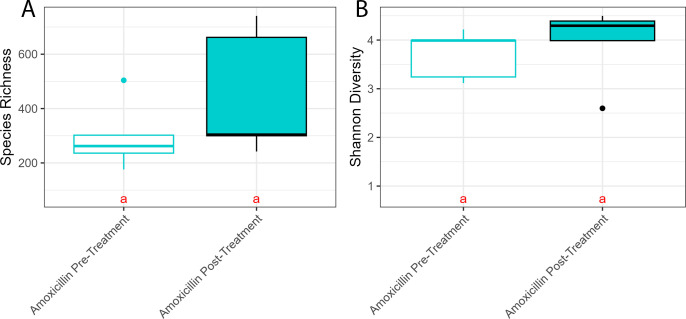
Alpha diversity of apparently healthy tissue pre- (white) and post-treatment with amoxicillin (blue) measured by (A) species richness and (B) Shannon diversity. Apparently healthy tissue was collected from CYBD colonies, approximately 10 cm beyond a CYBD lesion. Pretreatment samples were taken immediately before amoxicillin treatment, and post-treatment samples were taken from the same area 2 days after treatment. Letters denote significant differences determined by *post-hoc* Tukey Honestly Significant Difference test.

Species richness and Shannon diversity of the apparently healthy tissue did not significantly differ between samples from treated or untreated areas or through time (Table S3). There were no significant differences in either alpha diversity metric when comparing the apparently healthy tissue of the amoxicillin-treated area pre- and post-treatment with amoxicillin (Fig. 9, richness Tukey *post-hoc* comparison *P* = 0.350, Shannon Tukey *post-hoc* comparison *P* = 0.779; Table S3). In addition, there were no differences in the alpha diversity between the pre- and post-treatment time points for untreated apparently healthy tissue (richness Tukey *post-hoc* comparison *P* = 0.868, Shannon Tukey *post-hoc* comparison *P* = 0.692; Table S3) and for the lesion tissue from either the amoxicillin-treated or -untreated area (Fig. S5, richness Tukey *post-hoc* comparison *P* = 0.703, Shannon Tukey *post-hoc* comparison *P* = 0.527; Table S3).

**Fig 10:**
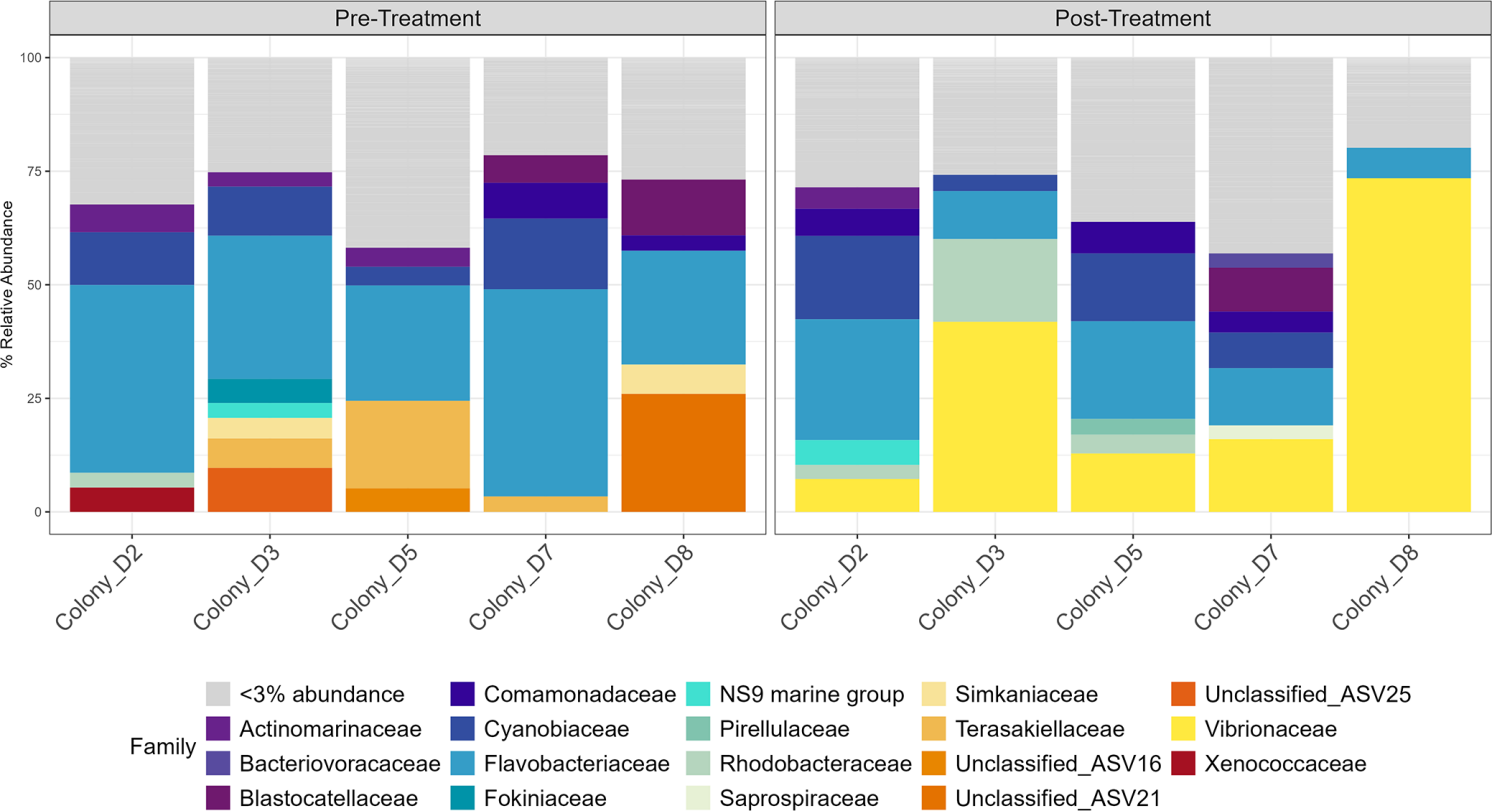
Relative abundance of bacterial families in the apparently healthy tissue samples pre-and post-treatment. Apparently healthy tissue was collected from CYBD colonies, approximately 10 cm beyond a CYBD lesion. Pretreatment samples were taken immediately before amoxicillin treatment, and post-treatment samples were taken from the same area 2 days after treatment. Displayed are families with a relative abundance over 3%. Families with relative abundance under 3% were grouped and represented in gray.

**Fig 11:**
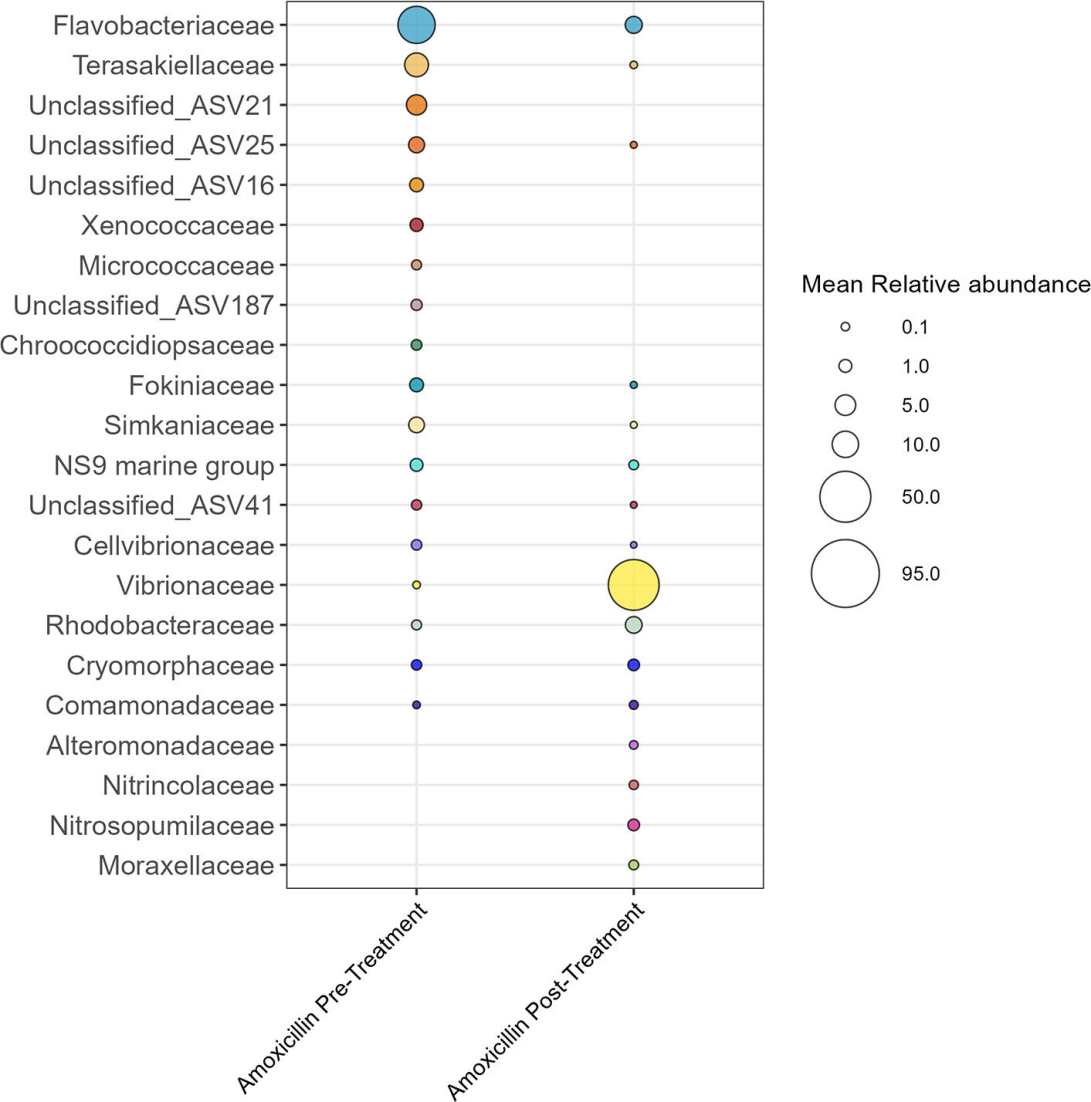
Differentially abundant amplicon sequence variants (ASVs) glommed by family and their relative abundances in the apparently healthy tissue pre-and post-treatment. Relative abundance was calculated for all of the ASVs in the amoxicillin data subset, and the top 100 abundant taxa were identified. Significant ASVs of the top 100 abundant taxa were determined by corncob analysis. Differentially abundant ASVs were glommed by family, due to low resolution at lower taxonomic ranks. Apparently healthy tissue was collected from CYBD colonies, approximately 10 cm beyond a CYBD lesion. Pretreatment samples were taken immediately before amoxicillin treatment, and post-treatment samples were taken from the same area 2 days after treatment.

Visualization of the relative abundances (mean ± standard error) supported the previous beta diversity results that treatment with amoxicillin shifted the microbial composition of the apparently healthy tissue (Fig. 10). This change was characterized by a large increase in *Vibrionaceae* relative abundance from 0.078% pretreatment to 30.30% ± 12.31% post-treatment (Fig. 10). The five most abundant bacterial families in the amoxicillin pretreatment samples were *Flavobacteriaceae* (33.78% ± 4.17%)*, Cyanobiaceae* (8.99% ± 2.40%)*, Terasakiellaceae* (6.70% ± 3.24%), *Blastocatellaceae* (3.91% ± 2.37%), and *Actinomarinaceae* (3.20% ± 0.94%). Post-treatment with amoxicillin, the most abundant families were *Vibrionaceae* (30.30% ± 12.31%)*, Flavobacteriaceae* (15.59% ± 3.67%)*, Cyanobiaceae* (9.47% ± 3.10%), Rhodobacteraceae (6.03% ± 3.06%), and Comamonadaceae (3.84% ± 1.28%). There were 45 ASVs that significantly differed between pre and post-treated apparently healthy samples, with 23 significantly positively enriched pretreatment and 22 positively enriched post-treatment (Fig. S6). Pretreatment ASVs belonged to 18 families, and post-treatment ASVs belonged to 16 families. A large proportion of the differentially abundant ASVs were present in one treatment but not in the other as some ASVs were knocked out after treatment and others were introduced (Fig. 11). Of note, relative abundances of the families *Vibrionaceae* and *Rhodobacteriaceae* increased post-treatment. Two *Vibrionaceae* ASVs were significantly positively enriched post-treatment, ASV3 and ASV40. ASV40 was present in small amounts in the apparently healthy tissue pre-treatment; however, ASV3 was completely absent pre-treatment and was the predominant *Vibrionaceae* ASV post-treatment. The lesion tissue was not colonized by *Vibrionaceae* even when amoxicillin was administered nearby (Fig. S7). The untreated samples were similar at both time points and similar to the pretreatment amoxicillin samples (Fig. S8).

## DISCUSSION

CYBD severely impacts colonies of the major Caribbean reef-building species, *Orbicella faveolata,* and microbiome studies are needed to not only understand the mechanism behind infection but also to determine how mitigation strategies like antibiotic treatments affect microbial communities. We evaluated the microbial communities of healthy control colonies, apparently healthy tissue from colonies with CYBD, and CYBD lesion tissue. Our results determined that the bacterial community of apparently healthy tissue on CYBD colonies was similar to those within lesion tissue, which may indicate an in-between or systemic disease state caused by dysbiosis. The disease mitigation strategy tested here, a firebreak and application of an amoxicillin-laced ointment, did not effectively halt CYBD lesion progression. Microbial compositions of the lesion tissues near treated and untreated areas did not significantly differ; however, amoxicillin treatment resulted in colonization of *Vibrionaceae* in the apparently healthy tissue, which was adjacent to the treatment area.

### Microbial compositions differed between healthy control colonies and those with CYBD

The present study found a difference between the bacterial communities of the healthy control tissue on healthy colonies of *O. faveolata* compared to the apparently healthy tissue on CYBD-diseased colonies. In addition, there was no difference between apparently healthy tissue and lesion tissue on CYBD-affected colonies. Unexpectedly, there were no differences between healthy controls and lesion tissue, although the ordination plots suggested these tissue types were largely separated within multivariate space (Fig. 2). A lack of statistical power due to low sample size and high levels of dispersion among sample types likely contributed to this lack of a statistically significant difference. Similar to our study, the microbiome of *O. faveolata* with CYBD in Puerto Morelos, Mexico, differed between the healthy control tissue and the apparently healthy tissue on diseased colonies and no difference between apparently healthy tissue and lesion tissue, but did find a difference between lesion and control tissues (28).

Alpha diversity was higher in samples from the apparently healthy and lesion tissue of colonies with CYBD than in tissue samples from the healthy controls. This was similar to the results from Closek et al. (28), which found that CYBD colonies had higher bacterial richness compared to healthy control colonies. However, our results differ as Closek et al. (28) found that apparently healthy tissue on diseased colonies had higher richness than lesion tissue, and we found no difference between the tissue types on the CYBD colonies (28). The apparently healthy tissue in our study was sampled approximately 10 cm from the lesion, whereas apparently healthy tissue sampled by Closek et al. (28) was sampled 30–90 cm from the lesion. The higher bacterial richness and Shannon diversity in tissue from CYBD colonies may have resulted from an increase in dysbiosis associated with the disease state (11). Our combined diversity results support the hypothesis put forth by Closek et al. (28) that, although sampled from the apparently healthy tissue, microbial compositions of CYBD colonies are distinct from those of healthy colonies. Similar to that of the lesion tissue, the microbial composition of apparently healthy tissue may be an in-between disease state that affects the tissue at least 10 cm from the lesion and potentially beyond. *Flavobacteriaceae* was the most abundant taxon in all three tissue types, which is consistent with previous literature that found it to be dominant in the coral surface mucus layer in both healthy and CYBD lesion tissues in a study conducted at Media Luna Reef, La Parguera, PR (48), and in healthy *O. faveolata* across four locations in the Caribbean (49). In the present study, ASVs from this family were significantly more enriched in CYBD colonies compared to healthy controls. There were only six families of ASVs that were significantly enriched in the healthy controls compared to the CYBD colonies. These included *Commondeaceae, Cyanobacteriaceae, Streptococcaceae, Pasteurellaceae, Cellvibrionaceae,* and *Terasakiellaceae*. It is possible that these bacterial families or their members are positively associated with a healthy microbiome of *O. faveolata*. Notably, *Terasakiellaceae and Comamonadaceae* were the second- and third-most abundant families in healthy samples, respectively, indicating a positive association with the healthy microbiome of *O. faveolata* (49, 50)

*Cyanobiaceae* was the bacterial family with the second-highest abundance in both apparently healthy and lesion tissue types and was fourth-most abundant in the healthy control. This family appeared in one colony on Mermaid Reef in Great Abaco, Bahamas, a reef that is resistant to bleaching (50), and was present in the disease core of *Acropora* in Florida (38). In the present study, it was significantly enriched in CYBD colonies and appeared in higher relative abundances in the apparently healthy tissue. *Rhodobacteraceae* was the third-most abundant family in the lesion tissue and has previously been enriched in corals with white band disease and implicated in SCTLD (38, 51–53).

In the present study, the CYBD colonies were characterized by greater familial diversity of differentially abundant ASVs compared to healthy controls. While members of the bacterial family *Vibrionaceae* were present in both healthy and CYBD colonies, ASVs from this family were slightly more enriched in diseased colonies. Taxa from several other families were more enriched in tissue from diseased colonies than those in the *Vibrionaceae* family. This may suggest that CYBD is not caused by a singular pathogenic agent and may be a consequence of dysbiosis or associated with some other type of pathogenic agent. Additional bacterial taxa, like those in the *Cyanobiaceae* family, that were present in healthy *O. faveolata* colonies but had higher relative abundances in diseased colonies could be considered potential opportunistic pathogens that thrive in the microbial communities of stressed, diseased coral. The bacterial families presented here were not ubiquitously associated with CYBD bacteria documented in previous studies or identified consistently within the healthy microbiome of *O. faveolata*. Indeed, there seems to be considerable variation in microbial communities present from both healthy and CYBD-affected *O. faveolata* in the literature (28, 48–50). This could be due to many reasons, including differences between the mucus, tissue, and skeletal compartments; geographical differences; or seasonal differences (54–56).

### Effect of amoxicillin treatment on CYBD progression and host microbial communities

Differences in microbial composition were investigated pre- and post-treatment with amoxicillin and among sample types post-treatment (amoxicillin treated, untreated, and healthy control). We hypothesized that the microbiome would change in response to amoxicillin and that the applied treatment may shift the microbial signature to resemble that of the healthy control tissue. Instead, we observed that apparently healthy tissue near areas treated with amoxicillin was significantly different from healthy controls.

There was no difference between the microbial communities of the lesion tissue near areas treated with amoxicillin and those of the lesion tissue near untreated areas for any of the alpha or beta diversity metrics. This is likely because the majority of the antibiotics applied in the trench between the lesion and the apparently healthy tissue was placed more on the side of the apparently healthy tissue than on the lesion side in an effort to treat the disease ahead of the lesion area.

Treatment with amoxicillin significantly changed the microbial communities of the apparently healthy tissue 2 days after the application. Previous studies have observed a decrease in bacterial richness and abundance following antibiotic treatment applied to healthy corals and those with other diseases (31, 57, 58). However, our results showed more dispersion in the post-treatment samples and no significant difference in the alpha diversity. While species richness and Shannon diversity pre- and post-treatment did not significantly differ, the distribution and composition of species varied after treatment. It is important to note that the aforementioned studies used a variety of other antibiotics, but not amoxicillin, as no published studies to our knowledge have documented the effect of amoxicillin on the coral microbiome, even though it has been used to effectively treat SCTLD (33). It is also important to note that prior studies (31, 57, 58) were performed *ex situ,* while the present study was performed *in situ*.

Amoxicillin application led to a shift in dominance by taxa in the family *Vibrionaceae*. One study on *Porites astreoides* in Curaçao followed the fate of corals that had been treated with a combination of nine antibiotics, none of which included amoxicillin, in aquaria and then returned to the reef (57). They observed that the aquaria corals treated with antibiotics remained healthy and had a lower overall abundance of bacteria than those that remained *in situ*. However, once they were returned to the field, the antibiotic-treated corals were colonized by opportunistic bacteria such as *Vibrionaceae* and rapidly deteriorated, while the control corals did not (57). An *ex situ* study on *Pocillopora* originally from Panama-treated corals with a combination of the antibiotics ampicillin, streptomycin, and ciprofloxacin and observed a shift toward a higher abundance of *Vibrionaceae* after antibiotic treatments (58). A different study of *Acropora* in aquaria reported that ampicillin and paromomycin sulfate knocked out *Vibrio* from the microbiome and halted lesion progression in white band disease, while gentamicin and metronidazole did not (31). A recent review concluded that most *Vibrio* spp. showed resistance to many common antimicrobials, including β-lactams antibiotics such as amoxicillin (59). The observed shift toward *Vibrionaceae* post-treatment could be due to the antibiotics reducing the abundance of the core members of the bacterial community, thus opening up a niche that could then be colonized by opportunistic bacteria, in this case *Vibrionaceae* (11).

Past research has identified four species of *Vibrio* as putative pathogens of CYBD by culturing bacteria isolated from affected corals and then inoculating them into healthy corals to initiate disease (23). The *Vibrio* spp. strains isolated were referred to as YB36, YBM23, YBFL3122, and YBFLG2A and displayed a close sequence homology with *V. alginolyticus* and *Vibrio parahaemolyticus*. It is theorized that these species work as a consortium to cause disease as inoculation with one strain alone is not enough to induce CYBD symptoms. However, the role of *Vibrionaceae* in CYBD is still under investigation as other studies chronicling CYBD have reported *Vibrio* inconsistently present in diseased samples, *Vibrio* only in healthy samples, or *Vibrio* species being present in both healthy and diseased samples (27, 28). It is important to note that *Vibrionaceae* is a large bacterial family that includes well-known pathogens but largely comprises nonpathogenic species including important symbionts (60, 61), and more studies targeting the role of this family and specific *Vibrio* species within the coral holobiont are needed.

While treatment with amoxicillin resulted in a large increase in the relative abundance of family *Vibrionaceae* in the nearby apparently healthy tissue, the nearby lesion tissue did not see a similar shift. This could be due to the majority of the antibiotics being applied over the lip of the trench on the side of the apparently healthy tissue. Disease progression was measured over 6 months and compared between treated lesion areas and untreated lesion areas. The average monthly progression rates of lesions near treated and untreated areas were 0.45 cm/month and 0.52 cm/month, respectively, which corresponded with the value (0.6 cm/month) reported by Cervino et al. (18). Overall, we found that amoxicillin was unsuccessful at halting lesion progression in CYBD. The lack of therapeutic efficacy of amoxicillin and an increase in the putative pathogen *Vibrionaceae* without a difference in lesion progression could suggest this family plays less of a role in CYBD than previously thought and may be yet another opportunistic bacteria taking advantage of a disturbance event. It is also possible that *Vibrionaceae* is a secondary pathogen or coinfection with CYBD, as has been suggested for stony coral tissue loss disease (62).

Administration of broad-spectrum antibiotics must be approached with care as there are associated risks, such as increasing the potential for antibiotic resistance (63). The effect of antibiotics on coral microbiomes should be evaluated further to study coral disease dynamics, how treatments affect holobiont constituents, and identify possible risks of implementation. This study was limited to sampling the bacterial community, and future studies should explore if amoxicillin treatment affects other members of the coral holobiont, such as the *Symbiodiniaceae* community. It is also of interest to investigate potential microbiome shifts when utilizing other treatments for coral diseases such as CoralCure D and E, which have antibacterial and antiviral properties and were successful in halting black band disease progression (36), or probiotics, which have shown promise in treating stony coral tissue loss disease (64). Developing treatments to coral disease remains a vital and time-sensitive field of study, and evaluating the microbiome in the context of these diseases and their treatments is critical for identifying an effective therapy to combat coral diseases.

### Conclusion

Caribbean yellow band disease poses a significant threat to reef-building corals in the Caribbean. The pathogenic agent is still unknown, and mechanical treatments, to date, have lacked long-term efficacy. Our study determined bacterial families associated with healthy or CYBD-affected *O. faveolata* and documented how those communities changed after treatment with amoxicillin. Both lesions and apparently healthy tissue from diseased colonies had higher alpha diversity than healthy control colonies, suggesting potential dysbiosis. In addition, the bacterial communities of apparently healthy tissue on diseased colonies significantly differed from healthy controls and were similar to that of lesion tissue, suggesting that apparently healthy tissue on diseased colonies represents an in-between or systemic disease state. Treatment with amoxicillin was not effective at halting lesion progression over the 6-month study period. However, amoxicillin significantly altered the composition of the microbiome within apparently healthy tissue adjacent to the treatment. The most notable microbial shift after antibiotic treatment was the colonization with the family *Vibrionaceae,* which is considered to be a putative pathogen for CYBD. However, since lesion progression did not worsen in response to this colonization, and next-generation sequencing-based studies do not consistently attribute *Vibrionaceae* to a disease phenotype, it is possible that this family has less of a role in CYBD than previously thought. Future studies are needed to further decipher aspects of the host-microbiome interactions in addition to the bacterial compartment. This study highlights the importance of validating the efficacy of coral disease treatments and understanding the impacts on the microbiome in order to properly assess risks associated with large-scale implementation of disease mitigation strategies.

## Data Availability

The data and code are available at GitHub: https://github.com/AlexiPearson-Lund/Evaluating-the-Effect-of-Amoxicillin-Treatment-on-the-Microbiome-of-O.-Faveolata-with-CYBD. The 16s rRNA sequences are available at NCBI under the BioProject accession number PRJNA1257591.

## SUPPLEMENTAL MATERIAL

Supplemental material aem.02407-24-s0001.docx Tables S1 to S4; Figures S1 to S8.

ASM does not own the copyrights to Supplemental Material that may be linked to, or accessed through, an article. The authors have granted ASM a non-exclusive, world-wide license to publish the Supplemental Material files. Please contact the corresponding author directly for reuse.
