# Supplementary material for: Evaluating the effect of amoxicillin treatment on the microbiome of Orbicella faveolata with Caribbean yellow band disease
Source: Appl Environ Microbiol. 2025 Jun 12;91(7):e02407-24. doi: 10.1128/aem.02407-24 (PMC12285242; doi:10.1128/aem.02407-24)
Supplement: Supplemental material — Tables S1 to S4; Figures S1 to S8. [file aem.02407-24-s0001.docx]

# **Supplemental Material**

**Abbreviation Key:**

Les – Lesion

UT – Untreated

AH – Apparently Healthy

Amox = Amoxicillin treated

**Table S1. Table of Reads Retained Through the Bioinformatics Pipeline**

| Initial number of reads | 38,659,556 |
| --- | --- |
| Number of reads after filter and trim step in DADA2 | 23,587,579 |
| Number of reads after removing any ASVs that were considerably off target length | 22,060,073 |
| Number of reads after chimeras were removed | 21,033,399 |
| Number of reads after the pruning step to remove 0 taxa and taxa in less than 4 samples (equated to 952 reads) | 21,032,447 |
| Number of reads after chloroplasts (2,958,698 ) removed | 18,073,749 |
| Final number of reads after mitochondria (327,050) removed | 17,746,699 |

**Table S2. Beta Diversity Metrics. Full PERMANOVA results for all datasets and results of the multiple PERMANOVA tests with Bonferroni adjusted p-values.**

| **Data Set** | **Variable** | **DF** | **F** | **R^2^** | **p-val** | **Multiple Comparisons** | **Adjusted**  **p-value** |
| --- | --- | --- | --- | --- | --- | --- | --- |
| Entire Data Set | Treatment  Time. Point  Treatment: Time | 2  1  2 | 2.27  1.827  1.034 | 0.122  0.033  0.037 | 0.001***  0.032*  0.377 |  |  |
| Pre and Post: AH Untreated, AH Amoxicillin, Control | Treatment  Time.Point  Treatment:Time | 2  1  2 | 2.435 | .152 | 0.001*** |  |  |
|  |  |  | 1.360  0.917 | .043  0.057 | 0.093  0.599 |  |  |
| Pre and Post - AH Untreated and AH Amoxicillin | Treatment  Time.Point  Treatment:Time | 1  1  1 | 1.804  1.752  1.201 | 0.087  0.084  0.058 | 0.023*  0.018*  0.215 |  |  |
| Pre: AH Untreated, AH Amoxicillin, Control | Treatment | 2 | 1.656 | 0.216 | 0.012* | Control - UT | 0.339 |
|  |  |  |  |  |  | Amox - UT | 0.912 |
|  |  |  |  |  |  | Amox - Control | 0.030* |
| Post: AH Untreated, AH Amoxicillin, Control | Treatment | 2 | 1.695 | 0.221 | 0.014* | Control - UT | 0.252 |
|  |  |  |  |  |  | Amox - UT | 0.129 |
|  |  |  |  |  |  | Amox - Control | 0.009* |
| Pre + Post: Control | Time.Point | 1 | 0.486 | 0.057 | 0.937 |  |  |
| Pre +Post : Untreated Apparently Healthy | Time.Point | 1 | 0.577 | 0.0673 | 0.882 |  |  |
| Pre + Post: Amox Apparently Healthy | Time.Point | 1 | 2.441 | 0.234 | 0.008* |  |  |
| Post : AH Untreated, Untreated Lesion, Control | Sample.Type | 2 | 1.548 | 0.147 | 0.031* | UT AH - UT Les | 0.801 |
|  |  |  |  |  |  | Control - UT Les | 0.198 |
|  |  |  |  |  |  | Control – UT AH | 0.045* |
| Post: Untreated Lesion, Amoxicillin Lesion | Treatment | 1 | 1.143 | 0.125 | 0.235 |  |  |
|  |  |  |  |  |  |  |  |
|  |  |  |  |  |  |  |  |
|  |  |  |  |  |  |  |  |

**Table S3. Alpha Diversity Metrics. Results of ANOVAs and Tukey HSD post-hoc multiple comparison tests of Species Richness and Shannon Diversity.
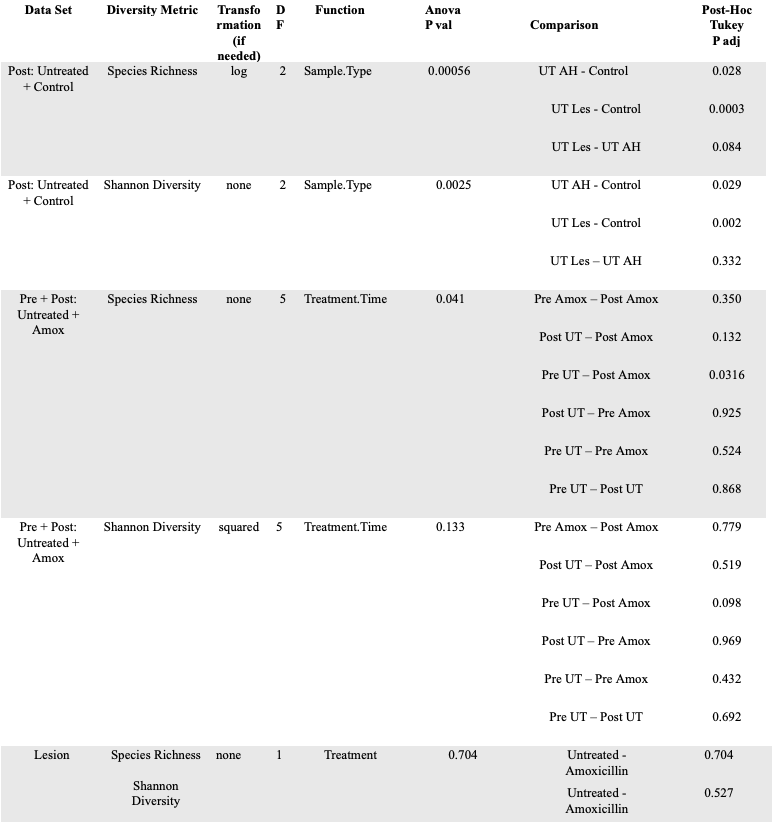
**

**Table S4. Additional Beta Diversity Metrics. Betadisper results of homogeneity of variance.**
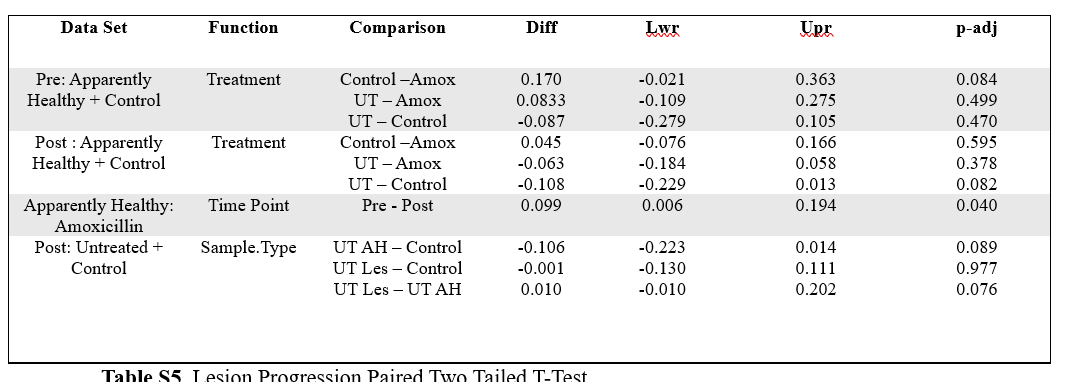


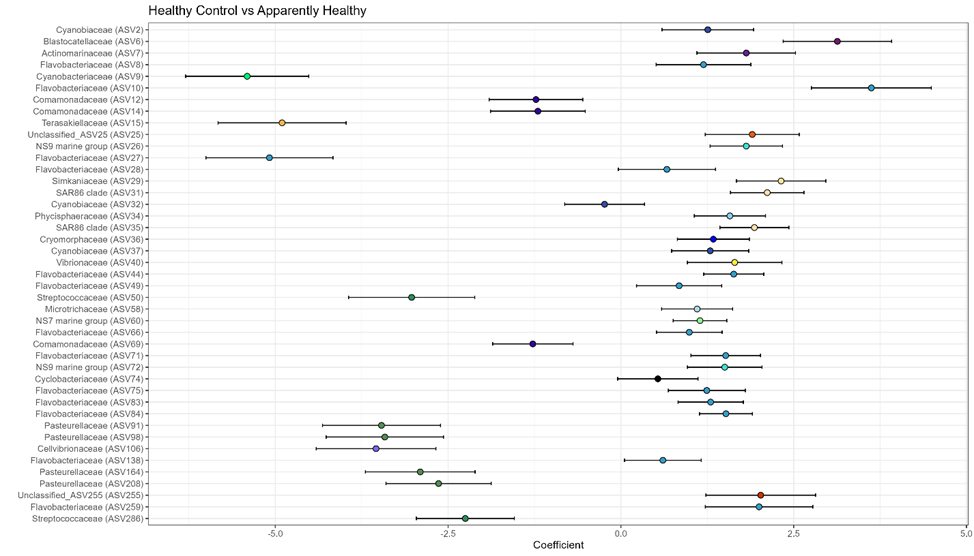


**Figure S1. Corncob differential abundance analysis of healthy control vs apparently healthy tissue from CYBD colonies.** ASVs that were significantly enriched in the healthy control tissue from healthy control colonies showing no signs of disease are represented to the left of the 0 coefficient, and those that were significantly enriched in the apparently healthy tissue from CYBD colonies are represented to the right. Bacterial families are depicted on the y-axis with the specific ASV number in parenthesis. Healthy control colonies had no signs of Caribbean yellow band disease (CYBD) disease. Apparently healthy tissue was collected from CYBD colonies within tissue that appeared healthy and was 10 cm beyond a CYBD lesion. Only samples at the post-treatment time point were included in this analysis since lesion tissue was only sampled at that time point. None of the included samples were treated with antibiotics.


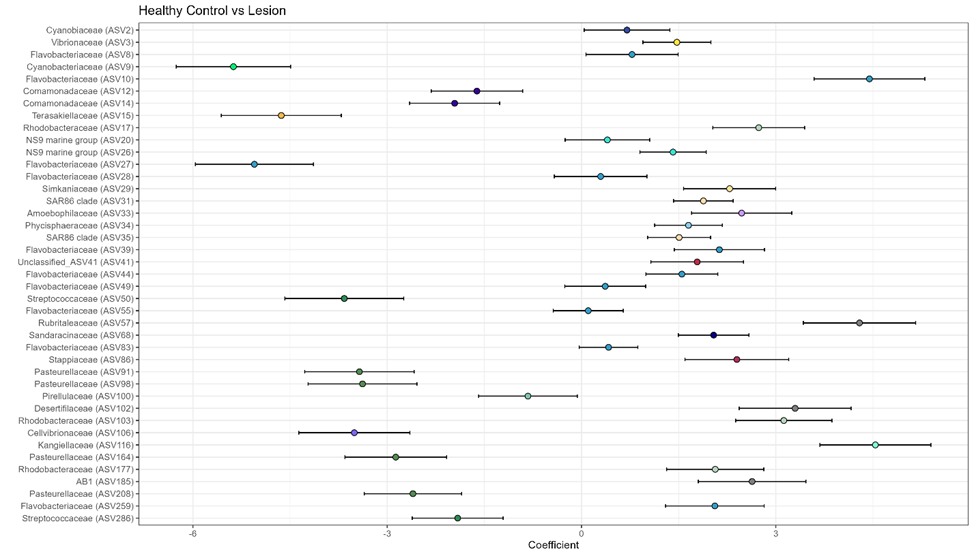


**Figure S2. Corncob differential abundance analysis of healthy control vs CYBD lesion tissue.** ASVs that were significantly enriched in the healthy control tissue from healthy control colonies showing no signs of disease are represented to the left of the 0 coefficient, and those that were significantly enriched in the CYBD lesion tissue are represented to the right. Bacterial families are depicted on the y-axis with the specific ASV number in parenthesis. Healthy control colonies had no signs of Caribbean yellow band disease (CYBD) disease. Lesion tissue was within the CYBD lesion. Only samples at the post-treatment time point were included in this analysis since lesion tissue was only sampled at that time point. None of the included samples were treated with antibiotics.


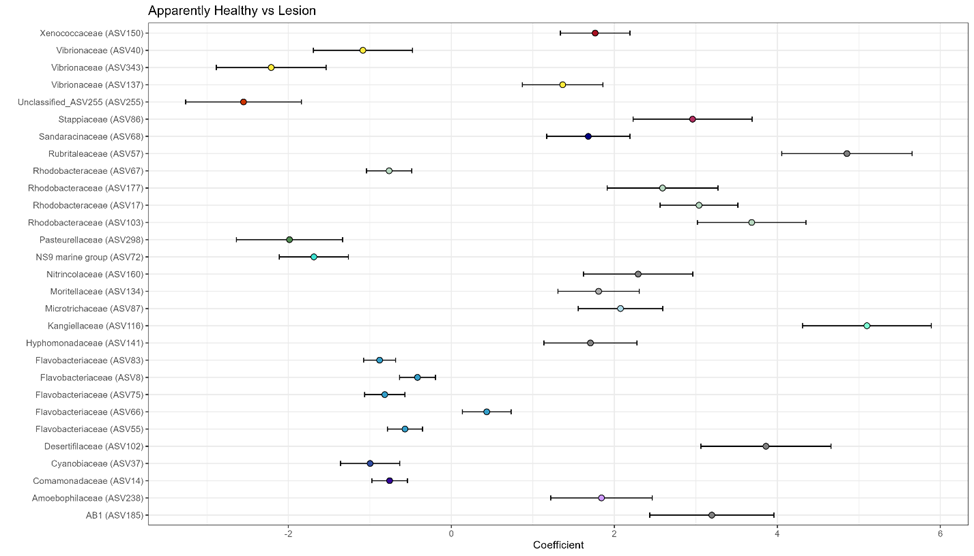


**Figure S3. Corncob differential abundance analysis of apparently healthy tissue from CYBD colonies vs CYBD lesion tissue.** ASVs that were significantly enriched in the apparently healthy tissue from CYBD colonies are represented to the left of the 0 coefficient, and those that were significantly enriched in the CYBD lesion tissue are represented to the right. Apparently healthy tissue was collected from CYBD colonies within tissue that appeared healthy and was 10 cm beyond a CYBD lesion. Bacterial families are depicted on the y-axis with the specific ASV number in parenthesis. Lesion tissue was within the CYBD lesion. Only samples at the post-treatment time point were included in this analysis since lesion tissue was only sampled at that time point. None of the included samples were treated with antibiotics.


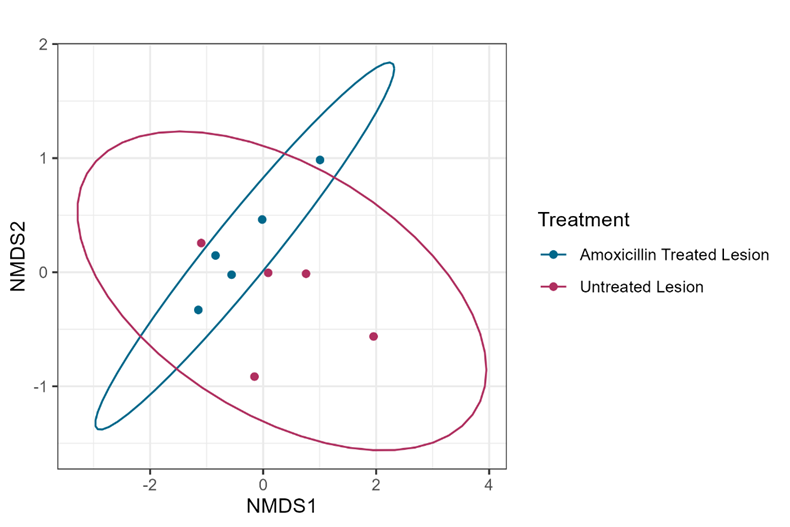


**Figure S4. Non-metric multidimensional scaling analysis (NMDS) of the Bray-Curtis Distances calculated on the amplicon sequence variants (ASVs) of CYBD lesion tissue either from amoxicillin treated or untreated lesion areas.** Tissue from CYBD lesions that were from either untreated (maroon) or treated lesion areas (dark blue) were sampled. All lesion tissue samples were taken at the post-treatment time point. Points represent samples. Untreated lesion (n=8) and Amoxicillin Lesion (n=8). Ellipses represent the 95% confidence interval.


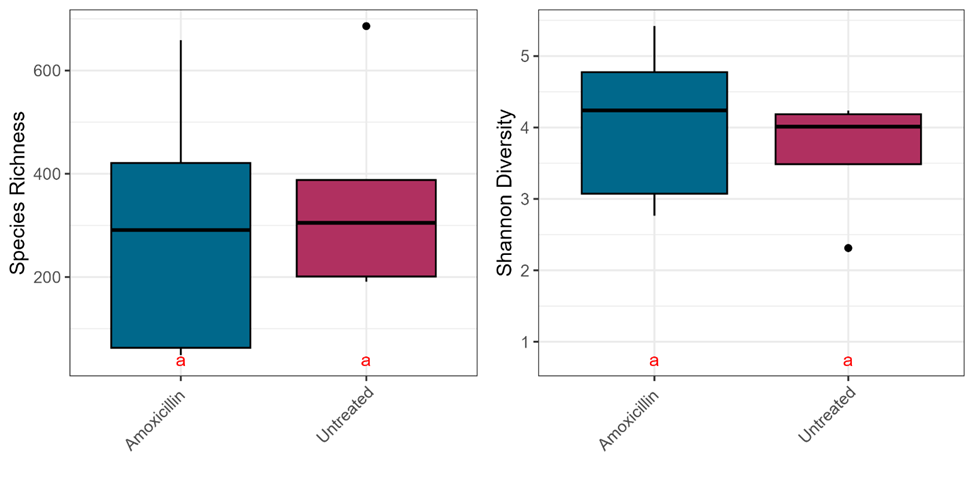


**Figure S5. Alpha diversity of lesion tissue from either treated or untreated lesion areas measured by species richness and Shannon diversity.** Letters denote significant differences determined by post-hoc Tukey Honestly Significant Difference test. CYBD lesions were sampled after amoxicillin treatment (dark blue) or from untreated (maroon) lesion areas. Only samples at the post-treatment time point were included in this analysis since lesion tissue was only sampled at that time point.


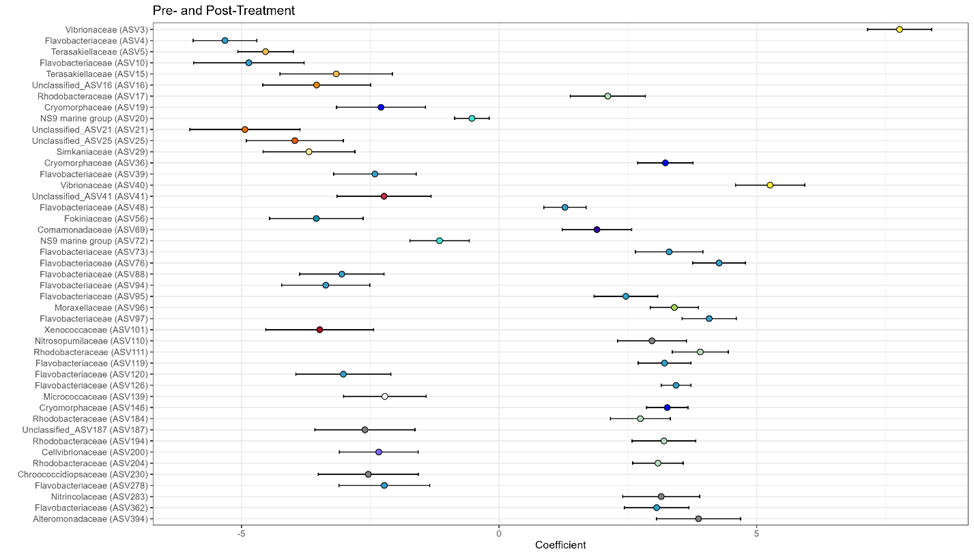
**Figure S6. Corncob differential abundance analysis of apparently healthy tissue pre- and post-treatment with amoxicillin.** ASVs that were significantly enriched in the apparently healthy tissue on CYBD colonies pre-treatment with amoxicillin are represented to the left of the 0 coefficient, and those that were significantly enriched in the apparently healthy tissue post-treatment with amoxicillin are represented to the right. Bacterial families are depicted on the y-axis with the specific ASV number in parenthesis.

**
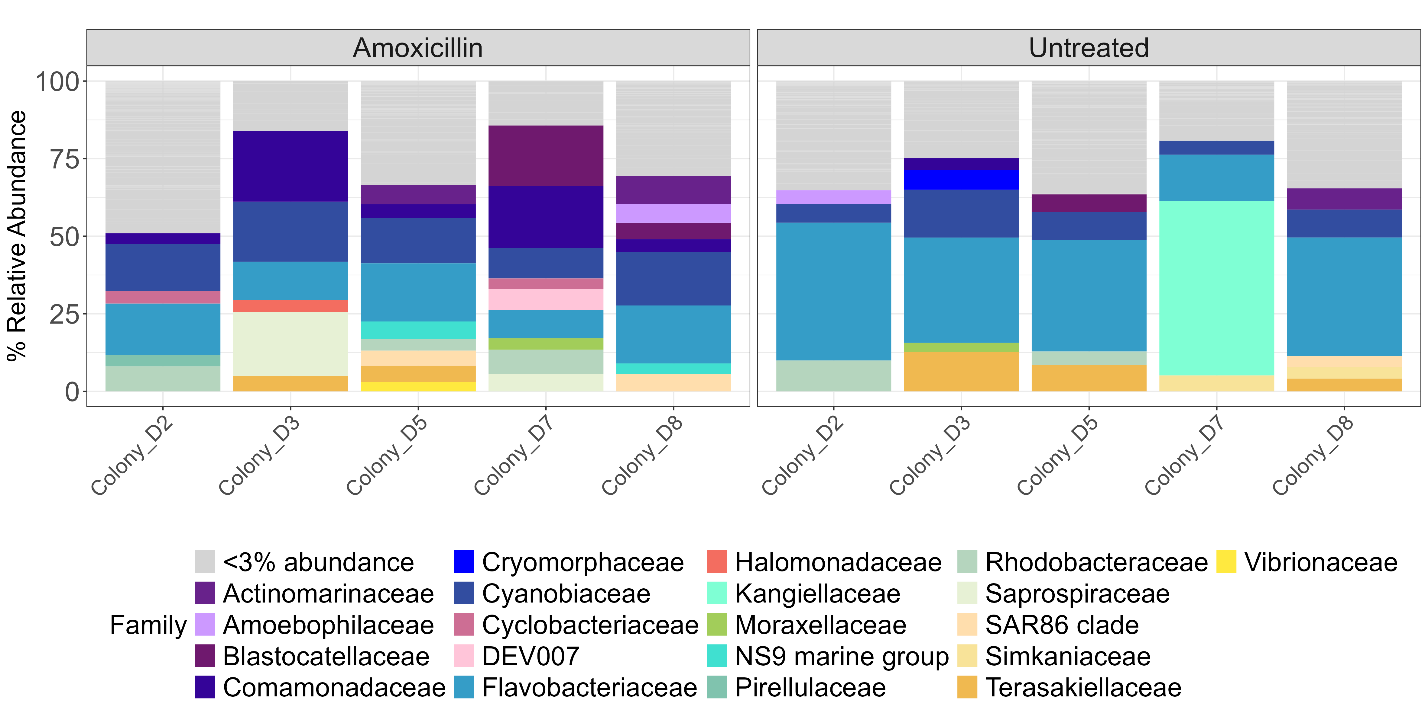
**

**Figure S7. Relative abundance of bacterial families in the lesion tissue**. CYBD lesions were sampled from amoxicillin treated areas or from untreated areas. Only samples at the post-treatment time point were included in this analysis since lesion tissue was only sampled at that time point. Displayed are families with a relative abundance over 3%. Families with relative abundance under 3% were grouped and represented in gray.

**
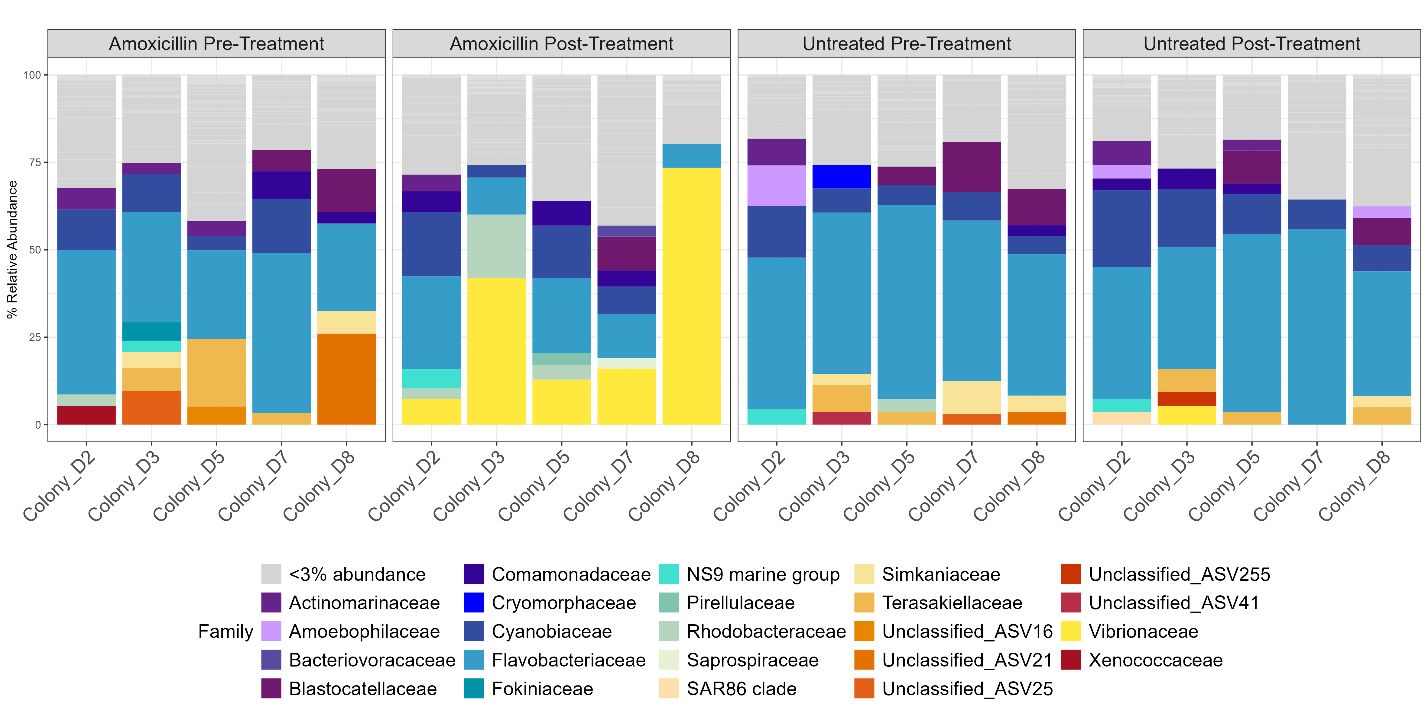
**

**Figure S8. Relative abundance of bacterial families in apparently healthy tissue either treated or untreated with amoxicillin both pre- and post treatment.** Apparently healthy tissue was collected from CYBD colonies within tissue that appeared healthy and was 10 cm beyond a CYBD lesion. Pre-treatment samples were taken immediately before amoxicillin treatment and post-treatment samples were taken from the same area two days after treatment. Untreated samples never received antibiotics. Displayed are families with a relative abundance of over 3%. Families with relative abundance under 3% were grouped and represented in gray.
